# Nuclear RNA Sequencing of the Mouse Erythroid Cell Transcriptome

**DOI:** 10.1371/journal.pone.0049274

**Published:** 2012-11-29

**Authors:** Jennifer A. Mitchell, Ieuan Clay, David Umlauf, Chih-yu Chen, Catherine A. Moir, Christopher H. Eskiw, Stefan Schoenfelder, Lyubomira Chakalova, Takashi Nagano, Peter Fraser

**Affiliations:** 1 Department of Cell and Systems Biology, University of Toronto, Toronto, Ontario, Canada; 2 Laboratory of Nuclear Dynamics, The Babraham Institute, Babraham Research Campus, Cambridge, United Kingdom; 3 Progeria Research Team, Centre for Cell and Chromosome Biology, Biosciences, School of Health Sciences and Social Care, Brunel University, West London, United Kingdom; University College London, United Kingdom

## Abstract

In addition to protein coding genes a substantial proportion of mammalian genomes are transcribed. However, most transcriptome studies investigate steady-state mRNA levels, ignoring a considerable fraction of the transcribed genome. In addition, steady-state mRNA levels are influenced by both transcriptional and posttranscriptional mechanisms, and thus do not provide a clear picture of transcriptional output. Here, using deep sequencing of nuclear RNAs (nucRNA-Seq) in parallel with chromatin immunoprecipitation sequencing (ChIP-Seq) of active RNA polymerase II, we compared the nuclear transcriptome of mouse anemic spleen erythroid cells with polymerase occupancy on a genome-wide scale. We demonstrate that unspliced transcripts quantified by nucRNA-seq correlate with primary transcript frequencies measured by RNA FISH, but differ from steady-state mRNA levels measured by poly(A)-enriched RNA-seq. Highly expressed protein coding genes showed good correlation between RNAPII occupancy and transcriptional output; however, genome-wide we observed a poor correlation between transcriptional output and RNAPII association. This poor correlation is due to intergenic regions associated with RNAPII which correspond with transcription factor bound regulatory regions and a group of stable, nuclear-retained long non-coding transcripts. In conclusion, sequencing the nuclear transcriptome provides an opportunity to investigate the transcriptional landscape in a given cell type through quantification of unspliced primary transcripts and the identification of nuclear-retained long non-coding RNAs.

## Introduction

While the full complexity of mammalian transcriptomes has yet to be characterized, it is clear that far more transcription occurs than can be accounted for by protein-coding genes alone [1], [2], [3]. Transcription of both coding and non-coding RNA (ncRNA) by the eukaryotic RNA polymerase II (RNAPII) complex requires the co-operation of numerous factors to control polymerase recruitment and promoter escape, transcriptional initiation, elongation and termination (reviewed in [4]). Each of these distinct stages represents a potential point at which gene expression can be regulated. For example, several studies have revealed that RNAPII is present in higher levels at the 5′ end of many eukaryotic genes compared to the downstream regions of the gene [5], [6], [7], [8], leading to the idea of promoter-proximal ‘pausing’ or ‘stalling’ of the transcription complex. Using a global nuclear run-on-sequencing assay (GRO-Seq) stalled RNAPII associated with the 5′ end of genes was found to be engaged in the production of sense transcripts downstream of the promoter as well as antisense RNA upstream of the promoter [9]. Such transcription may play an important role in transcriptional interference and bystander effects which have been reported for mammalian genomes [10], [11], [12], [13], [14].

A study of global genome folding revealed that the active and inactive portions of the genome are individually segregated [15] consistent with compartmentalisation of transcription in mammalian nuclei. On a smaller scale, the three-dimensional folding of chromatin in the nucleus is an important factor in regulating gene expression in a tissue-specific manner [16]. In the developmentally regulated murine β-globin locus (*Hbb*), tissue-specific chromatin loops form between expressed genes and the locus control region (LCR) located approximately 50 kb upstream [17], [18], [19]. Similar chromatin loops have now been identified and implicated in the regulation of numerous other gene loci, including: the α-globin locus (*Hba*), *Th2*, *MHC*, *IgH*, *Igκ*, *HoxB1*, *CFTR*, and olfactory receptor genes [20]–[27].

The multitude of processes that influence regulation of transcription impose challenges on the analysis of the transcriptome. In this study we analyse transcriptional output and RNAPII association in adult mouse anemic spleen erythroid cells by generating genome-wide nuclear RNA and RNAPII chromatin-association [28] profiles. We demonstrate that nucRNA-Seq provides a representative description of nascent transcription at erythroid-expressed genes. Through comparative analyses, we show that high and low transcriptional output correlate with particular patterns of polymerase occupancy. With integration of publicly available data, we identify putative regulatory regions and ncRNAs that are stably retained in the nucleus of erythroid cells.

## Results

### Nuclear transcriptome generation and validation

**Figure 1:**
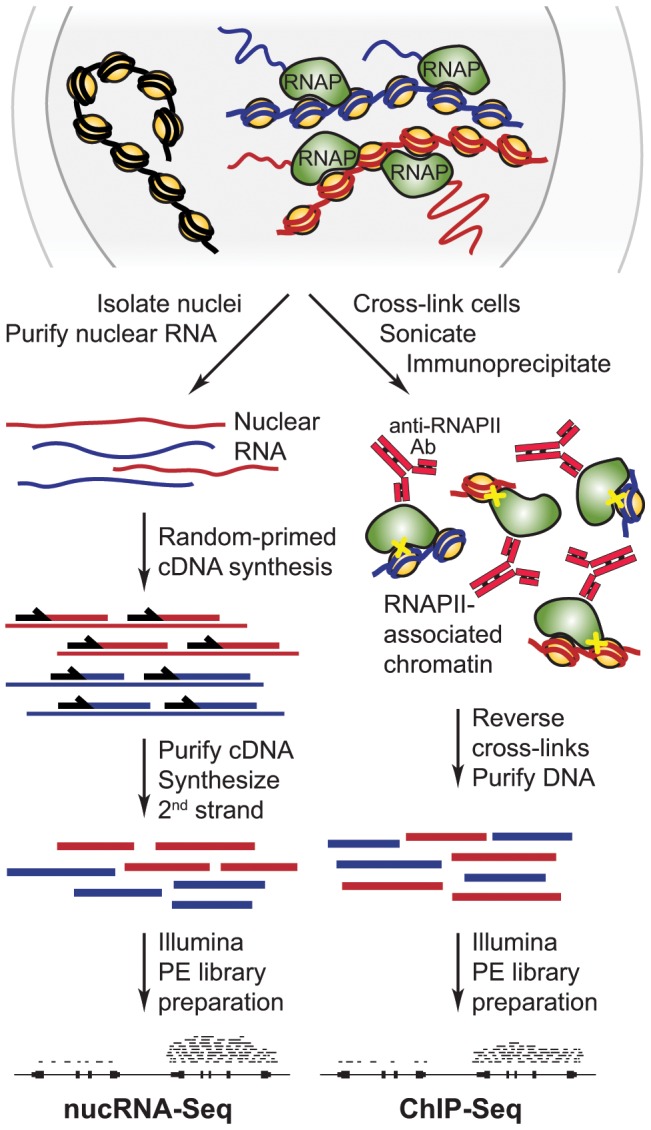
Outline of the experimental strategy. The nuclear transcriptome as well as RNAPII-associated genomic sequences of actively transcribing cells are analysed by nucRNA-Seq and RNAPII ChIP-Seq, respectively, as indicated. Top: schematic representation of transcription in the nucleus: four transcribing RNAPII complexes depicted as green shapes are associated with two chromatin fibres, DNA shown in red and blue, respectively; a third chromatin region, which is not being transcribed, is shown with DNA in black; histone complexes are yellow circles, nascent transcripts are shown as thin wavy lines, colours corresponding to chromatin. The nucRNA-Seq procedure is outlined on the left; purified nuclear RNA from the two transcribed regions is shown as wavy or straight lines colour-coded as above, DNA is depicted as thicker lines, random primers are black arrows, a putative genomic region with aligned Illumina paired-end (PE) tags signifies nucRNA-Seq data. The RNAPII ChIP-Seq procedure is outlined on the right; immunoprecipitated RNAPII-associated nucleosomes are depicted and colour-coded as above with cross-links as yellow crosses, anti-RNAPII antibodies are shown as red Y shapes, purified DNA is represented by thick lines, a putative genomic region with PE tags signifies RNAPII ChIP-Seq data.

**Figure 2:**
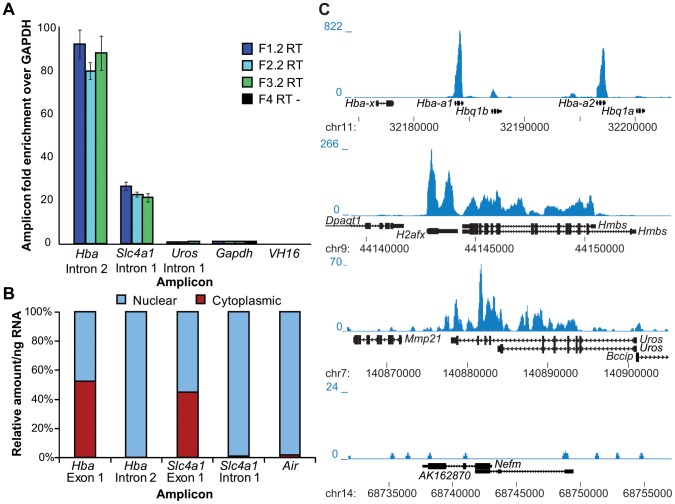
Validation of nuclear RNA material and sequence coverage at selected genes. A) Quantitative PCR validation of transcript representation for cDNA samples used for construction of nucRNA-Seq libraries (F1.2, F2.2 and F3.2, F4 is the RT- sample for F1.2) relative to the housekeeping gene *Gapdh* was confirmed. Error bars depict one standard deviation calculated from three technical replicates. B) Quantitative PCR validation of nuclear/cytoplasmic fractionation. Nuclear and cytoplasmic RNA was reverse transcribed using random primers to generate cDNA. Absolute quantities of specific gene regions were determined in these samples by real-time PCR using genomic DNA standard curves. The relative amount in each fraction per ng of RNA is depicted. We found exonic sequences were distributed between the nuclear and cytoplasmic fractions while intronic sequences were found almost exclusively in the nuclear fraction. Furthermore, we found *Air* ncRNA almost exclusively in the nuclear fraction. **C**) Shown are selected genes: erythroid-specific (*Hba cluster, Hmbs, Uros*), ubiquitous (*H2afx*) and a brain-specific gene, *Nefm* that is not expressed in erythroid cells. Nuclear RNA sequence coverage is shown in blue. All genomic regions are depicted from centromere to telomere and the 5′ end of the gene is marked by the gene name.

RNA-Seq libraries are usually generated from poly(A) positive RNA isolated from intact cells and thus reflect the steady-state levels of mRNA present in the cell population [29]. As we wanted to investigate nascent transcriptional output, we isolated intact nuclei, purified total nuclear RNA and used random primers for reverse transcription (RT) to generate cDNA representative of nuclear RNA (Figure 1). Initial quantitative real-time RT-PCR (RT-qPCR) quality control performed on nuclear RNA confirmed that *Hba*, *Slc4a1* and *Uros*, known erythroid-expressed genes were represented, while a non-transcribed region of the IgH locus (VH16) was not detected (Figure 2A). To assess the level of enrichment for nuclear RNA species, we performed RT-qPCR on the nuclear and cytoplasmic RNA fractions using exonic and intronic primer pairs. As expected, we found exonic sequences were distributed between the two fractions while intronic sequences were found almost exclusively in the nuclear fraction (Figure 2B). Furthermore, we found the *Air* long non-coding RNA almost exclusively in the nuclear fraction in agreement with the finding that *Air* RNA is retained in the nucleus [30], [31].

We generated a nuclear transcriptome for adult mouse anemic spleen erythroid cells by sequencing our validated nuclear RNA (nucRNA-Seq) using the Illumina paired-end sequencing protocol (Figure 1). We obtained greater than ten million aligned sequence pairs from three replicate nucRNA double-stranded cDNA libraries. As expected, we observed high nucRNA-Seq coverage (sequence representation) at strongly expressed erythroid-specific genes, including the adult α-like globin (*Hba-a1*, *Hba-a2*) and β-like globin genes (*Hbb-b1* and *Hbb-b2*), the erythrocyte membrane protein band 3 (*Scl4a1*) and hydroxymethylbilane synthase (*Hmbs*, selected genes shown in Figure 2C). More moderately expressed genes such as the heme pathway member *Uros* (uroporphyrinogen III synthase) had lower coverage, while the silent, brain-specific gene *Nefm* was not enriched above the surrounding intergenic background coverage.

We calculated the number of reads per kilobase of gene length per million mapped reads (RPKM) at mouse Ensembl genes for our three replicate nucRNA-Seq libraries (Table S1) [32]. A comparison of RPKM values in our three biological replicate libraries indicated that relative transcript abundance was reproducible between samples (Spearman's rho >0.8, Figure S1). In addition we compared the observed coverage and reproducibility for 48 randomly selected nucRNA-Seq enriched regions to RNA levels in two independent nuclear RNA preparations by RT-qPCR. We observed a significant association between RT-qPCR results and nucRNA-Seq coverage (Figure S2). Considering the biological replicate nucRNA-Seq libraries individually, we observed highly reproducible coverage for these regions, however as the association with our RT-qPCR data was seen to be stronger for the combined nucRNA-Seq data than any of the 3 individual experiments, the nucRNA-Seq data was considered as one dataset for the remaining analyses.

### NucRNA-Seq coverage reflects primary transcription levels

To confirm that nucRNA-Seq is indicative of raw transcriptional output, we compared exonic and intronic coverage to demonstrate that the sequence data was derived mainly from unprocessed, immature transcripts. In a hypothetical, completely unspliced transcript, we would expect the mean exonic and intronic coverage depths to match, with removal of introns resulting in an exonic bias (high coverage of exons relative to introns). As a proof of concept for this hypothesis, we first compared our nucRNA-Seq dataset to a poly(A) enriched RNA-Seq dataset from a similar cell type. The G1E cells are committed erythroid progenitor cells derived from *Gata1*-null mouse ES cells [33]. These cells undergo terminal differentiation upon restoration of *Gata1* expression. This is achieved upon treatment with estradiol (E2) in the G1E-ER4 subline which contains an estrogen-activated *Gata1*-estrogen receptor transgene [34], [35]. We initially compared the exonic and intronic coverage in our nucRNA-Seq data with the intronic and exonic coverage in G1E and G1E-ER4+E2 RNA-Seq datasets [36]. As expected the G1E and G1E-ER4+E2 RNA-Seq mapped mainly to exons (85% and 95% exonic respectively) however our nucRNA-Seq library showed a strong bias toward intronic reads as introns are generally much larger than exons (36% exonic).

**Figure 3:**
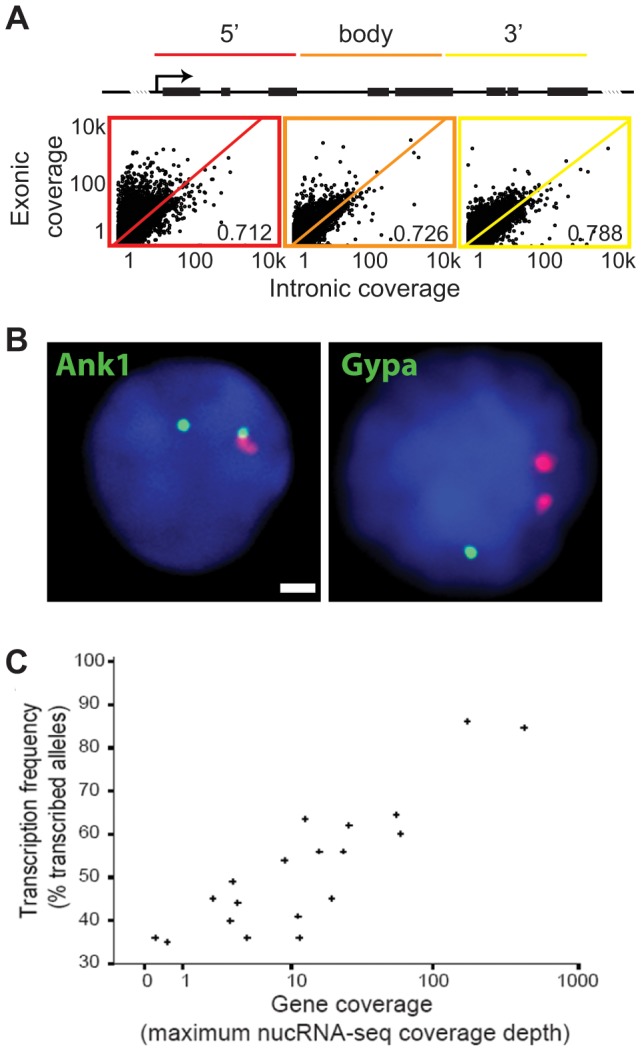
Sequencing nuclear RNA reflects primary transcription at erythroid-expressed genes. A) Exonic vs intronic coverage for annotated genes in the 5′ (red), body (orange) and 3′ (yellow) regions by splitting each gene into equal thirds. B) Examples of RNA FISH signals for *Ank1* and *Gypa* shown in green, *Hbb-b1* is shown in red, nuclear DAPI staining is shown in blue, scale bar = 1 µm. C) Transcription frequency determined by RNA FISH compared to gene coverage in nucRNA-Seq data. We found a significant log-linear association between the transcription frequency determined by RNA FISH and the maximum nucRNA coverage depth (r_s_ = 0.820, 95% CI [0.582, 0.928], p<0.01).

To provide further evidence that we were capturing primary transcription, we next investigated the exonic and intronic coverage in more detail in our nucRNA-Seq library. As true sequence enrichment can be masked by bias introduced during alignment against a reference genome [37], [38], sequence coverage was normalised relative to a sequenced genomic input DNA library in order to assess true biological enrichment (Figure S3). Unless otherwise stated, all subsequent data is given as fold enrichment over input. Average nucRNA-Seq coverage at annotated genes shows a significant correlation between exonic and intronic regions (Figure 3A, r_s_ = 0.850, 95% CI [0.844, 0.855], p<0.01). Dividing each gene into 5′, body, and 3′ thirds, we observed the association between exonic and intronic coverage levels to increase in a 5′ to 3′ direction (5′ r_s_ = 0.712, 95% CI [0.702, 0.721], p<0.01; body r_s_ = 0.726, 95% CI [0.716, 0.734], p<0.01; 3′ r_s_ = 0.788, 95% CI [0.781, 0.794], p<0.01), with 5′ regions displaying a slight exonic bias (Figure 3A). Both of these observations are consistent with current models of co-transcriptional splicing [39] and with the conclusion that our nucRNA-Seq coverage represents nascent transcription.

In addition, we compared the average nucRNA-Seq coverage depth at 19 erythroid-expressed genes to the transcription frequency determined by RNA fluorescence in-situ hybridisation (FISH, Figure 3B, 3C) [40]. We used intronic probes to determine the number of cells in the population with actively transcribing alleles for any given gene. Transcription frequency was calculated as the percentage of expressed alleles in the population, i.e. those with detectable signals (Table S2). We found a significant log-linear association between the transcription frequency determined by RNA FISH and the maximum nucRNA coverage depth (r_s_ = 0.820, 95% CI [0.582, 0.928], p<0.01). This indicates that nucRNA quantity is reflective of the frequency at which primary transcription occurs in the cell population for erythroid-expressed protein-coding genes (Figure 3B, 3C).

Taken together, our results indicate that nucRNA-Seq data reflect *in vivo* primary transcript levels. We were interested to investigate how different the relative levels of primary transcripts were compared to total poly(A) positive mRNA. To do this we compared RPKM coverage over the first exon in our nucRNA-Seq data to RPKM coverage over the first exon in the G1E and G1E-ER4+E2 RNA-Seq data (Figure S4). While the correlation between the two RNA-Seq libraries was quite high (Spearman's rho = 0.88) correlation between nucRNA-Seq and RNA-Seq data was low (Spearman's rho = 0.25 and 0.30). Specifically we noted that coverage at exon 1 was more often overrepresented in the nucRNA-Seq data compared to the RNA-Seq data.

### RNAPII ChIP-Seq generation and validation

As our nucRNA-Seq data correlated with *in vivo* primary transcript levels we next wanted to investigate the relationship between nucRNA levels and RNAPII association throughout the genome. We performed chromatin immunoprecipitation (ChIP) of the large subunit of the polymerase complex (RPB1) phosphorylated at serine 5 (S5) of the carboxy-terminal domain (CTD). The RPB1 CTD consists of a heptapeptide repeat of the consensus sequence YSPTSPS [41] which is unphosphorylated during initial recruitment to promoters as part of a pre-initiation complex. In promoter-proximal regions the CTD is phosphorylated on S5 (S5P) which leads to recruitment of the capping enzyme [42], [43], [44], [45]. The S5P modification of the CTD acquired at the initiation phase of transcription persists throughout the transcription cycle as this polymerase form has been found associated throughout the body of transcribed and poised genes [46], [47], [48], [49]. In our ChIP experiments we therefore used an antibody that detects the S5 phosphorylated form of RNAPII. As part of our initial quality control we assayed fold enrichment in the RNAPII ChIP sample compared to the input sample by qPCR at a subset of expressed and non-expressed genes. As expected we found high levels of enrichment at erythroid-expressed genes and no enrichment at silent genes (Figure S5).

Sequencing of RNAPII ChIP (ChIP-Seq) using the Illumina paired-end sequencing protocol produced more than six million paired, aligned 36 bp sequences from each of the immuno-purified and genomic input fractions. As expected, and similar to the nucRNA-Seq coverage, RNAPII was associated with erythroid-expressed genes (Figure S6). As for nucRNA-Seq, we normalised the RNAPII ChIP-Seq to the sequenced input in order to assess true biological enrichment (Figure S3) [37]. At highly expressed genes (e.g. *Slc4a1*, Figure S6) we found sequence enrichment in the RNAPII ChIP-Seq data throughout the entire transcription unit. This enrichment was also identified throughout the *Slc4a1* transcription unit by qPCR (Figure S5).

In order to further validate observed coverage, fold enrichment over input was assessed by qPCR for 3 independent ChIP experiments and compared to our sequencing data at the same 48 randomly selected regions used to validate the nucRNA-Seq coverage. We observed a significant association between the fold enrichment assessed by qPCR and the RNAPII ChIP-Seq data, both for maximum coverage depth in the tested amplicon (r_s_ = 0.683, 95% CI [0.489, 0.812], p<0.01) and for average coverage depth (r_s_ = 0.668, 95% CI [0.477, 0.799], p<0.01) (Figure S7).

### Comparative analysis of nucRNA-Seq and RNAPII ChIP-Seq

**Figure 4:**
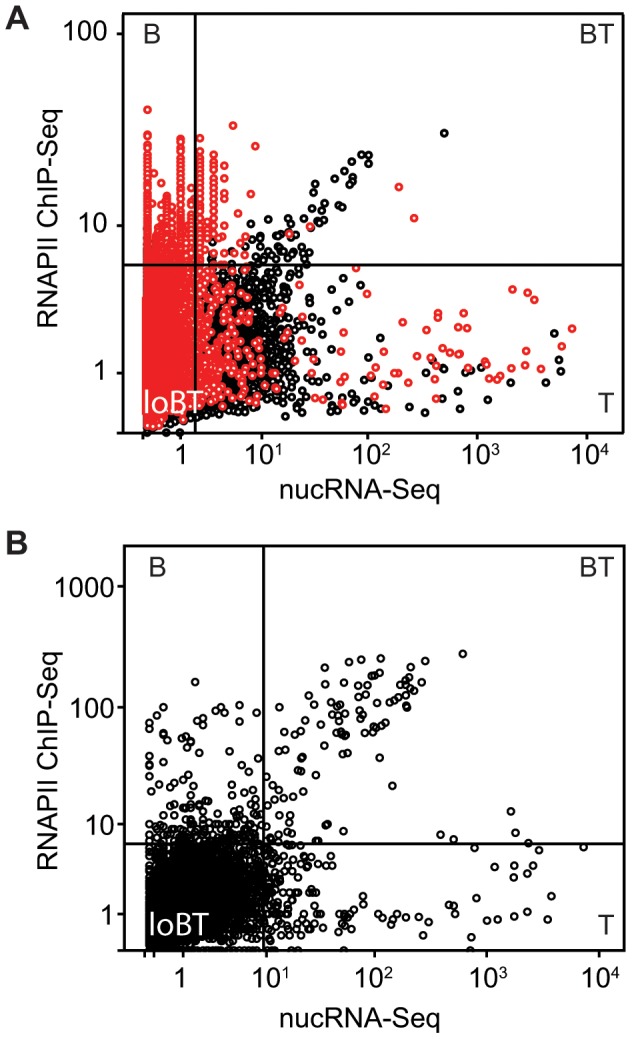
Comparison between RNAPII ChIP-Seq and nuclearRNA-Seq coverage. A) RNAPII vs nucRNA scores were calculated as the maximum coverage depth within non-overlapping 10 kb windows, normalised to the genomic input score. Threshold values for identifying highly enriched regions were calculated using the boxplot method (thresholds set as Q3+(1.5×IQR), where Q3 is the upper quartile limit and IQR the interquartile range) and are represented as black bars. Windows containing an annotated gene are depicted as black circles, windows lacking an annotated gene are depicted as red circles. Regions were classed as either being highly RNAPII-bound and transcribed (BT); highly transcribed, but with low RNAPII binding (T); or highly bound, but not highly transcribed (B); low levels of both RNAPII association and transcription (loBT). B) Scores were calculated for annotated genes only, as described above.

We next investigated the relationship between RNAPII association examined by RNAPII ChIP-Seq and transcriptional output assayed by nucRNA-Seq. Using a 10 kb window, we compared RNAPII ChIP-Seq and nucRNA-Seq coverage depth throughout the genome. We identified the highly enriched outliers in each dataset using the boxplot method (thresholds set as Q3+(1.5×IQR), where Q3 is the upper quartile limit and IQR the interquartile range). Using these thresholds we defined four classes of genomic sequences in our data; regions that were highly RNAPII-bound and transcribed (BT), bound by RNAPII but not highly transcribed (B), transcribed but not highly RNAPII-associated (T), and regions that were not highly RNAPII-associated or transcribed (loBT, Figure 4A). Regions of the genome falling into the BT and T categories frequently overlapped Ensembl genes (75 and 94% respectively). In contrast, genomic regions in the B class were less frequently associated with genic regions, with only 26.6% of the regions in this class overlapping with an Ensembl gene. In further data analysis we investigated RNAPII association and transcriptional output at the genic and intergenic regions of the genome separately.

### RNAPII association and nascent transcription at annotated genes

To explore the relationship between polymerase association and nascent transcription for genic regions, we re-defined the BT, B, T, and loBT classes in our data based on coverage of all Ensembl genes (NCBIM37, Figure 4B) rather than 10 kb windows. We identified 369 genes in the B category, 372 in the T and 191 genes in the BT categories, with the remainder (30480, genes ≤300 bp removed) showing lower levels of both polymerase association and transcription (loBT). These observations are consistent with current models which show very highly expressed genes to be in the minority [6], [7], [9].

Next we calculated the ratio of nucRNA-Seq: RNAPII ChIP-Seq maximum coverage depth as a measure of polymerase transcription efficiency. BT genes display a more efficient ratio of 0.936 compared to 0.394 for loBT genes (5% trimmed mean for each category) suggesting that the observed differences in transcriptional output are not simply explained by different levels of RNAPII association. Instead, it appears that polymerase associated with BT genes is producing RNA more efficiently. The T group displays the most efficient average nucRNA-Seq: RNAPII ChIP-Seq ratio of 12.7.

To probe whether there were any functional relationships behind the differences we observed in transcriptional behaviour, we compared the Gene Ontology (GO) term enrichments between the BT, T and B groups using the DAVID (Database for Annotation, Visualization and Integrated Discovery, Table S3) [50], [51]. As expected, BT was enriched in highly expressed genes and significantly enriched in erythroid functional categories as well as DNA replication and DNA packaging GO terms. Anemic spleen cells are rapidly dividing and would therefore require the expression of genes associated with DNA replication. The T category was significantly enriched in translation-related terms including; ribonucleoprotein complex and ribosomal proteins. The ribosomal protein genes in the T group may represent those with increased RNA stability as ribosomal proteins are tightly regulated both transcriptionally and post-transcriptionally to balance the production of ribosomal components [52], [53]. We also identified nucRNA coverage at the genes encoding *Terc* (vertebrate telomerase RNA), small nucleolar RNAs, signal recognition particle RNAs, micro RNAs, and small Cajal body-specific RNAs in the T group of genes suggesting this group is enriched for stable functional and structural RNAs. We later use this signature to identify intergenic regions encoding stable nuclear-retained transcripts (described below). Also included in this group were rRNAs and 7SK RNAs, which are transcribed by RNAPI and RNAPIII respectively and are therefore not expected to associate with RNAPII. B genes seem to be involved in stress responses (though the enrichments were not significant following multiple testing correction) and housekeeping functions (cell cycle, translation), which may point to “poising” of certain genes in readiness for anticipated functions.

**Figure 5:**
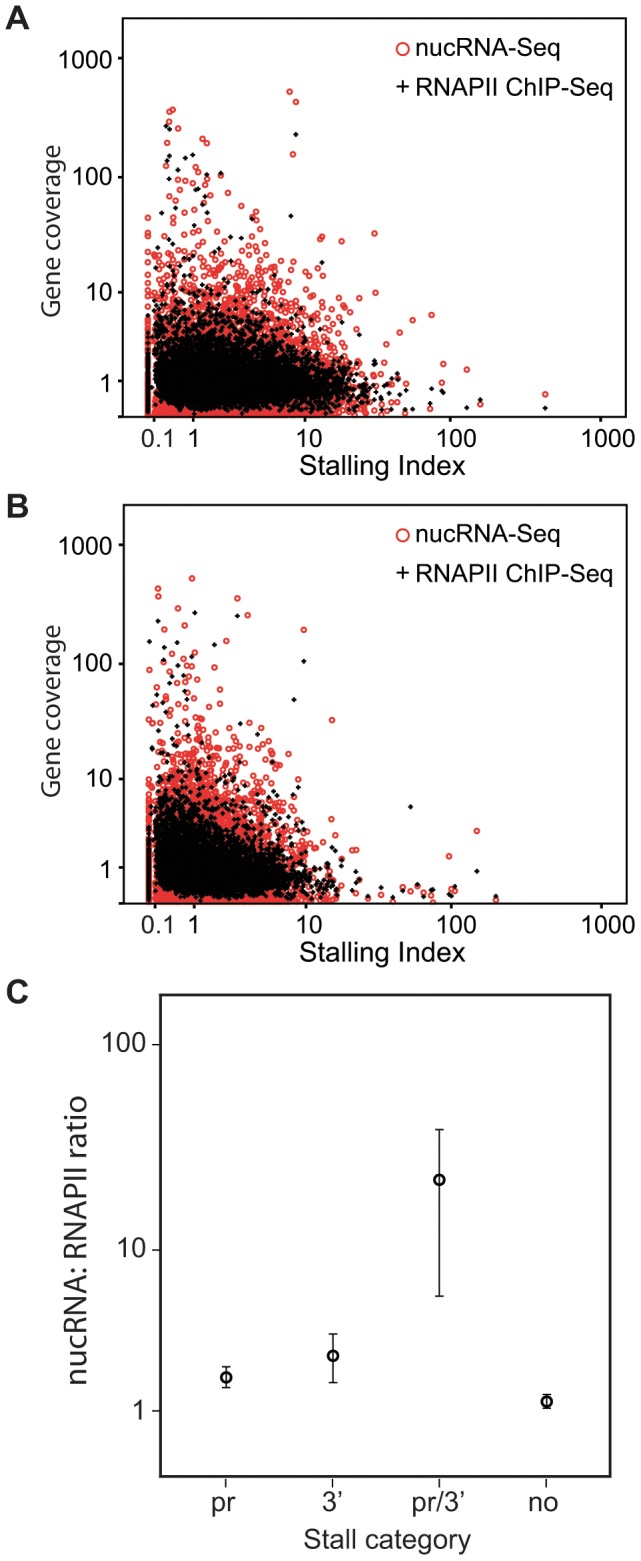
RNAPII peaks are associated with both the promoter and the 3′ end of genes. A) Promoter-proximal (±300 bp) stalling index plotted against RNAPII and nucRNA coverage at annotated genes. B) 3′ end (±300 bp) stalling index plotted against RNAPII and nucRNA coverage at annotated genes. C) nucRNA to RNAPII coverage ratio for the promoter (pr), 3′ end (3′) and double RNAPII peak (pr/3′) categories as well as at genes with low stalling indices at both ends (no).

As the GO analysis of the B category highlighted stress-response genes, including heat shock genes which are known to be poised for activation with polymerase stalled in the promoter-proximal region [54], [55], we calculated “stalling indices” for all annotated genes by identifying peaks of RNAPII in the promoter-proximal region [6], [8], [9], [56]. Similar to the work of Zeitlinger *et al* [8], we calculated the stalling index as the ratio of the maximum promoter-proximal (transcription start site, TSS ±300 bp) signal and the mean body signal, with a high ratio indicating “stalling”. Similar to previous findings [6], [8] we found a trend for genes with high promoter-proximal RNAPII peaks to have significantly lower levels of nucRNA coverage (p<0.001, Jonckheere-Terpstra test, Figure 5A). In fact, our data revealed an inverse correlation between total RNAPII coverage and promoter-proximal RNAPII peaks with the most highly RNAPII-associated genes having low stalling indices. This fits with our observation that the most highly expressed erythroid-specific genes (for example *Hba* and *Slc4a1* Figures S5 and S6) generally contained RNAPII associated throughout the entire transcription unit.

We also observed similar RNAPII peaks at the 3′ end of selected genes. We applied the same formula to calculate a “3′ end stalling index” for all annotated genes. Similar to the results for promoter-proximal RNAPII peaks, we found a significant trend for genes with high 3′ end stalling indices to have lower levels of nucRNA and RNAPII coverage in the gene body (p<0.001, Jonckheere-Terpstra test, Figure 5B). To further dissect patterns of occupancy, we compared promoter-proximal and 3′ end RNAPII peaks, identifying 300 genes with promoter, 300 genes with 3′ end, and 60 genes with both promoter and 3′ end RNAPII peaks (thresholds set at the 95^th^ percentile, Figures S8 and S9, Table S4). We observed that the “double RNAPII peak” genes have less polymerase in the body than other categories, but show equal if not higher levels of RNA compared to other categories. Examining the relationship between RNA and polymerase (only considering the body of the gene to avoid the identified RNAPII peaks at the 5′ and 3′ ends) we found that the double RNAPII peak group produces more RNA per polymerase than the other categories, with the ratio of RNA to polymerase being significantly higher (p<0.0001, Kruskal-Wallace ANOVA, Figure 5C). We conclude that transcription of a gene is clearly not a simple case of RNA production following polymerase binding, as patterns of RNAPII occupancy can correlate with transcription negatively (in the case of “stalling” at either end), or positively (in the case of the double RNAPII peak genes).

### Intergenic RNAPII is associated with regulatory regions

**Figure 6:**
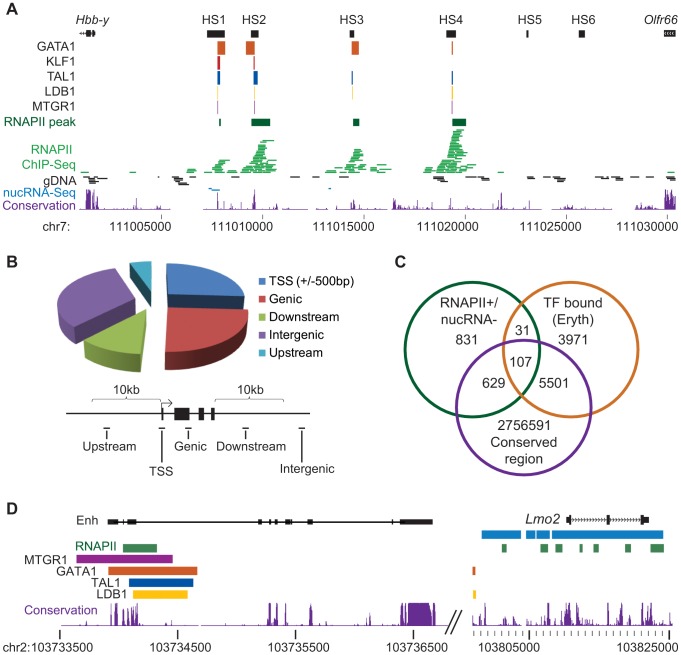
RNAPII is associated with enhancer regions. A) The *Hbb* (β-globin) LCR, located upstream of the *Hbb* genes, contains six characterized erythroid-specific DNase I hypersensitive sites (HS1-6). Peaks of RNAPII (green) identified using SISSRs overlapped HS1-4. Erythroid-expressed transcription factors have also been found associated with the LCR, overlapping the HS and RNAPII peaks. RNAPII ChIP sequences are shown in green, genomic DNA input sequences are shown in black and nucRNA sequences (only three in this region) are shown in blue. B) Distribution of RNAPII+/nucRNA- peaks relative to annotated genes. Roughly half of the RNAPII peaks identified by SISSRs are located in intergenic regions with 32.5% located more than 10 kb from an annotated gene (intergenic). C) Overlap of RNAPII+/nucRNA- peaks with erythroid-expressed transcription factors and conserved regions. D) An RNAPII+/nucRNA- peak 77 kb upstream of the *Lmo2* gene overlaps TF binding sites and is homologous to a validated enhancer identified in the human genome. Enhancer homology regions are indicated by black boxes joined by a line to delineate the human enhancer construct used in the generation of transgenic mice. NucRNA and RNAPII peaks surrounding the *Lmo2* gene are shown in blue and green respectively.

As previously mentioned, we found that intergenic regions of the genome tended to be associated with RNAPII in the absence of nuclear RNA (B group, Figure 4A). Previous studies have found that RNAPII in intergenic regions is associated with enhancer features [57], [58]. One of the most highly RNAPII-bound intergenic regions is located upstream of the *Hbb* genes and overlaps DNase I hypersensitive sites (HS) of the locus control region (LCR, Figure 6A). The *Hbb* LCR is a well characterized enhancer, required for high-level β-globin gene expression and has been shown to be in close physical proximity with the active *Hbb* genes, forming a chromatin loop [17], [19], [59]. In erythroid cells the HS of the *Hbb* LCR are bound by several transcription factors as well as RNAPII [35], [60], [61], [62], [63]. We observed very little nucRNA in the LCR region suggesting the associated polymerase is transcribing only at very low levels compared to transcription at expressed genes [64].

Taking the *Hbb* LCR as a prototypical example, we investigated other enhancer features at the regions associated with RNAPII but not nucRNA in erythroid cells. To delineate these regions we identified peaks in RNAPII binding using the SISSRs (Site Identification from Short Sequence Reads) algorithm which was originally designed to identify transcription factor binding sites [65]. From the resulting 3118 RNAPII+ peaks we removed those found to overlap with regions of nucRNA coverage (maximum gap width 100 bp; minimum size 1000 bp, identified in SeqMonk, [66]), thereby excluding those peaks in RNAPII binding that were associated with moderate to abundant transcription. This narrowed our original candidate list to 1598 RNAPII+/nucRNA- regions (Table S5). 25.9% of these overlap a TSS (+/−500 bp), 24.9% are found within a gene body, a further 5.3% and 11.4% are positioned within 10 kb upstream and downstream of a gene, respectively. The remaining 32.5% are located in intergenic regions, more than 10 kb away from the nearest annotated gene (at a mean distance of 0.4 Mbp from the nearest transcribed gene, defined by overlap with a nucRNA enriched region, Figure 6B).

We next investigated these RNAPII+/nucRNA- regions for additional features associated with regulatory regions. To this end, we examined the evolutionary sequence conservation around our candidates, which has been shown to improve identification of regulatory modules [67], [68]. In several studies, intergenic sequences with high evolutionary sequence conservation have been found to have enhancer activity in the developing embryo [69], to demarcate the regulatory elements of the human HBB locus [70], and to identify regulatory motifs on a genome-wide scale [71], [72], supporting the idea that these regions have regulatory potential. We calculated the proportion of PhastCons [73] conservation scores greater than 0.8 in the 1 kb sequence surrounding RNAPII peak midpoints and found significantly higher conservation at RNAPII+/nucRNA- regions compared to random regions (Log Odds ratio = 0.19, p<0.01).

Regulatory potential has been shown to be best predicted when sequence conservation information is integrated with transcription factor binding information [68], [74], [75]. As further validation that the RNAPII+/nucRNA- regions represented regulatory elements, we retrieved transcription factor (TF) ChIP-Seq datasets for mouse erythroid cells and compared RNAPII+/nucRNA- peaks to regions associated with the transcription factors GATA1, KLF1, LDB1, TAL1, ETO2 and MTGR1 (summarised in Table S6; [35], [62], [63]). After alignment, we identified TF binding peaks using the SISSRs algorithm [65], and found RNAPII+/nucRNA- peaks significantly overlapped transcription factor binding sites (for all TFs combined, Log Odds ratio = 3.04, p<0.0001, Table S7, Figure 6C). In addition to finding enrichment in individual TF binding sites within our putative regulatory elements, we also found that several RNAPII+/nucRNA- regions were bound by multiple TFs. Restricting our candidate list to an ‘erythroid subset’ (138 regions) which overlapped erythroid-expressed TFs improved the observed sequence conservation in those regions (Log Odds ratio = 1.48, p<1.8E-08) which likely indicates regulatory function. Some notable examples include the TF-bound HS of the *Hbb* LCR, a TF-bound validated enhancer upstream of the *Lmo2* gene [76] as well as TF-bound regions upstream of the *Pim1* and *Klf3* genes (Figure 6D and Figure S10). We also found significant overlap with p300 (Log Odds ratio = 1.6006, p<0.0001) ENCODE ChIP-Seq peaks identified in MEL cells (Tables S6 and S7) [2]. Peaks of the histone acetyl transferase p300 have been shown to predict regions with enhancer function in other tissues [77], [78], [79].

A large proportion of our RNAPII+/nucRNA- peaks do not overlap with TF binding sites identified through the transcription factor ChIP-Seq data for mouse erythroid cells, suggesting that the current suite of data may not represent all the TFs important in regulating gene expression in erythroid cells. We sought to investigate the possibility that the remaining candidates may still identify TF-bound regulatory regions by conducting a supervised motif search within these regions using JASPAR [80] TF binding profiles and the Clover algorithm [81]. To validate our approach, we first confirmed that the expected motifs were identified *in silico* in the ‘erythroid subset’ of RNAPII+/nucRNA- peaks known to bind erythroid-expressed TFs based on the ChIP-Seq data available (Table S7). We did identify enrichment in the motifs for TAL1/GATA1 (raw Clover score 31.9, p<0.001), both of which are TFs in the ‘erythroid subset’ (Table S8). We also identified motifs for KLF4 (Clover 40.8, p<0.001) and NFYA (Clover 7.69, p = 0.001), both known to regulate gene expression in erythroid cells [82], [83]. As the binding matrix for KLF1 (not contained in the Jaspar database) is highly similar to the binding matrix for KLF4 [63] and KLF4 expression is lower (nucRNA 1.0 fold enriched over input) than that of KLF1 (nucRNA 4.5 fold enriched over input) it is likely that enrichment of KLF4 motifs represents sequences predominantly bound by KLF1 in erythroid cells. The remaining RNAPII+/nucRNA- peaks contained profiles for a number of erythroid-expressed TFs including SPI1 [84] (Clover 133.0, p<0.001) and ETS1 [85] (Clover 26.8, p<0.001) (Table S9). This inferred TF binding potential further demonstrates the efficacy of using RNAPII binding to identify potential regulatory regions [57].

This approach allowed us to infer the involvement of TFs for which ChIP-Seq data is not available, and showed that RNAPII+/nucRNA- peaks identify regions under selective pressure containing binding sites for multiple cell type-specific and basal TFs.

### NucRNA-Seq identifies stable, nuclear-retained long non-coding RNAs

In addition to the RNAPII-associated intergenic regions we also noticed that a number of intergenic regions are transcribed above background levels. Many of these transcribed intergenic regions appeared to be several kilobases in size, potentially representing long non-coding RNAs (lncRNAs). lncRNAs are emerging as mediators in the regulation of genome function, alongside and in combination with epigenetic and transcription factor-based mechanisms [86], [87], [88], [89]. Many lncRNAs appear to regulate gene expression, primarily at the level of transcription (e.g. *Air* or *Xist*) [31], [87], [88], [90], [91]. We hypothesised that a class of stable nuclear-retained RNAs could be identified from the nucRNA-Seq data as intergenic transcription units with promoters bound by relevant transcription factors.

To obtain as large an initial candidate cohort as possible, we used the ‘Contig Probe Generator’ feature of SeqMonk [66], to identify clusters of nucRNA-Seq reads in an unbiased manner. By inspection, highly expressed genes were best identified allowing for a maximum 2 kb gap size between nucRNA-Seq reads and excluding candidates below 4.5 kb. Applying these conditions to our data, we identified 6,429 semi-contiguous regions of RNA coverage which did not overlap annotated genes (from an initial list of 24,396). We observed that low expressed genes were better identified with a different parameter set (1 kb gap size, 2.5 kb minimum candidate size, and merging candidates separated by less than 5 kb), which when applied to our data identified a further 1,154 candidates, yielding a final list of 7,583 candidate regions. This initial list excluded regions which overlapped annotated coding regions, pseudogenes, ribosomal RNAs and micro RNAs.

We hypothesised that at least a subset of the candidates could represent stable, nuclear retained RNA species. We therefore refined the candidate list to focus on stable lncRNAs by identifying candidates with relatively high levels of nucRNA-Seq coverage compared to RNAPII ChIP-Seq coverage. These candidates would therefore be part of the intergenic “T” subset discussed earlier. Candidate coverage was quantified as the average coverage depth, normalized for candidate length and total number of reads; 305 candidates with a higher nucRNA-Seq to RNAPII ChIP-Seq coverage ratio were selected (Table S10). From these 305 candidates, 72 (23.6%) overlapped RefSeq annotated ncRNA features including *Malat1*, *5830416P10Rik*, *A130040M12Rik*, *Gm1995* and *Neat1*, 17 (5.5%) overlapped lncRNAs identified by Guttman *et al.* 2009 and 36 (12%) overlapped erythroid expressed lncRNA identified by Hu *et al.* 2011 [92], [93]. Interestingly, our candidates often consolidated a cluster of previously identified lncRNAs into a larger transcript, for example the *Neat1* transcript (lncRNA2, Figure S11) [92].

**Figure 7:**
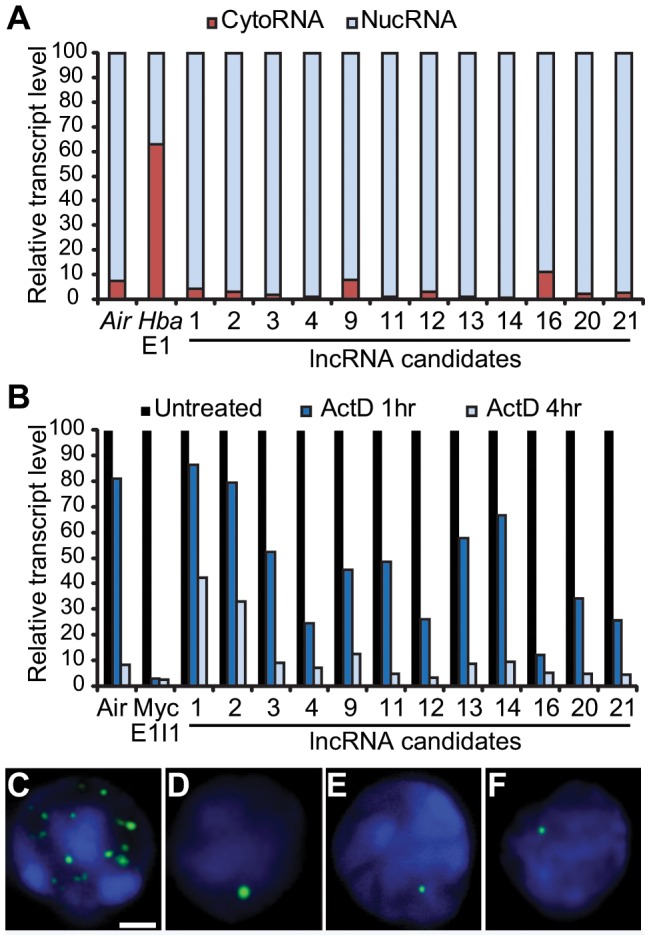
Transcribed intergenic regions correspond to long non-coding RNAs. A) Nuclear vs cytoplasmic distribution for lncRNA candidates determined by RT-qPCR. B) Stability of nuclear retained lncRNA candidates was assessed by treatment with ActD for 1 and 4 hrs. Transcript levels were determined by RT-qPCR. Intranuclear distribution of lncRNA candidates was determined by RNA FISH for: C) lncRNA1 (Malat1), D) lncRNA2 (Neat1), E) lncRNA9, and F) lncRNA11, scale bar = 2 µm.

We selected 12 candidates showing a variety of expression levels for further validation (Table S11). We first characterized these 12 candidate lncRNAs in terms of their nuclear confinement using RT-qPCR on nuclear and cytoplasmic RNA fractions. All 12 candidate tested were found to have a preferred nuclear localisation (Figure 7A). We then assessed the RNA stability of the 12 RNAs by qRT-PCR following Actinomycin D treatment to inhibit nascent transcription. All 12 proved to be more stable than the *Myc* primary transcript (Figure 7B) and several of the candidates showed transcript stability similar to *Air*. Lastly, strand prediction was done by comparing patterns of H3K4Me3 and H3K36Me3 histone modifications which are thought to identify promoter and gene body regions of transcription units, respectively. Data derived from ES cells was used [56] so not all candidates could be annotated (Table S11). Strand prediction based on histone modifications was confirmed by RNA FISH in all candidates. Furthermore, RNA FISH revealed a distribution into distinct nuclear foci (selected images shown in Figure 7C–F).

Candidate 1, which is by far the most highly expressed of all the candidates, corresponds to *Malat1* and was found distributed in multiple nuclear foci. *Malat1* (*Metastasis associated lung adenocarcinoma transcript 1*) is a lncRNA shown to be a post-transcriptional regulator of transcription in synaptogenesis [89], [94], though the high level of expression in erythroid cells suggests a wider role for this stable nuclear-retained lncRNA.

## Discussion

The recent explosion in the number of genome-wide datasets has greatly increased our appreciation of transcriptome complexity and regulation, particularly the role of polymerase distribution, intergenic regulatory elements and non-coding RNAs. Here we study transcriptional output in erythroid cells by sequencing nuclear RNA and chromatin bound by active RNA polymerase II. We show that nucRNA-Seq identifies mainly unspliced primary transcripts and is significantly different than poly(A)-enriched RNA-Seq. Then, we investigated the relationship between RNAPII occupancy and nucRNA output, identified intergenic regions of the genome associated with RNAPII which have characteristics of regulatory regions and identified novel, stable, nuclear-retained lncRNAs expressed in adult erythroid cells.

Our observations show that a generalized level of RNAPII occupancy is a poor predictor of expression levels for most transcription units, with only very highly expressed RNAPII-transcribed genes showing a correlation between RNAPII association and transcriptional output. These results suggest that polymerase occupancy is just one of potentially many factors influencing the level of transcription of chromatin templates. Peaks of RNAPII found in promoter-proximal regions have been suggested to represent paused polymerase and correlate with lower expression [6], [8]. Our analysis confirmed these observations in that RNAPII peaks at the 5′ end of genes generally correlated with lower expression of the genes. Furthermore, our results show that genes displaying RNAPII peaks at their 3′ ends are also poorly expressed. We also observed genes with RNAPII peaks within the gene body suggesting that other pause sites exist which may impede transcription. It remains to be determined whether or not these 3′ and internal RNAPII peaks actually represent engaged, paused polymerase. In accordance with these sites as polymerase pausing locations a study in *S. cerevisiae*, identifying the 3′ ends of nascent transcripts, using the NET-Seq (native elongating transcript sequencing) technique, identified numerous pause sites within genes [95].

We also found that accumulation of polymerase at the 5′ end of genes is not always associated with lower expression. In particular, genes featuring both 5′ and 3′ RNAPII peaks are more efficiently transcribed than genes with either peak alone. These peaks of RNAPII located within both the 5′ and 3′ regions of the “double RNAPII peak” genes may reflect a point of chromatin-chromatin interaction between these two regions allowing both locations to be captured in the RNAPII pull-down. Gene loop interactions between the promoter and 3′ end of inducible genes in *S. cerevisiae* have been associated with more rapid induction of transcription [96]. Our results indicating that genes displaying both 5′ and 3′ peaks of RNAPII are more efficiently transcribed suggest that similar gene loop interactions could occur at selected genes in higher eukaryotes and that these interactions contribute to increased gene expression.

Long range chromatin interactions are known to occur between regulatory regions and active genes [17], [19]–[27]. Our RNAPII ChIP-seq data identified intergenic regions bound by RNAPII, erythroid cell-expressed TFs and p300. This approach not only reveals regulatory regions by virtue of their TF binding properties, but potentially identifies the subset of regulatory regions physically associated with transcribing genes and as a result immunoprecipitated with the anti-RNAPII antibody. In agreement with this possibility, a subset of neuronal enhancers are bound by RNAPII [58]. However, in contrast to the neuronal study, we failed to detect enhancer-associated RNAs [58] in our dataset. We presume that these RNAs may not have been captured in our library preparation due to their size, stability or abundance. It has been shown that HS2 of the human *HBB* LCR has promoter activity and the entire LCR region is transcribed [97], [98], [99], [100], [101]. It is likely that the mouse LCR has similar properties and yet we did not identify significant levels of nucRNA in this region by nucRNA-Seq suggesting these transcripts are of relatively low abundance compared with the rest of the nuclear transcriptome. It should be noted that we cannot distinguish whether RNAPII is present at these regulatory regions as a result of their close association with the highly active *Hbb* gene, synthesis of short-lived LCR ncRNA, or both. A previous study has identified LCR transcripts and shown that RNAPII is present at the LCR in mouse embryonic stem cells which do not express any of the *Hbb* genes suggesting the LCR recruits RNAPII independently of and prior to *Hbb* gene transcription [102].

In sequencing the nuclear RNA pool we were able to identify stable, nuclear-retained lncRNAs. These RNA species were found to be enriched in the nuclear fraction and many are present at low levels. They are likely to be missed in approaches that isolate total RNA as the cytoplasmic RNA pool is larger than the nuclear RNA pool. In comparing to existing sets of lncRNAs identified from total RNA we found only limited overlap with our set indicating that by isolating the nuclear pool of RNA we were able to identify novel nuclear retained transcripts that are masked by the cytoplasmic pool in other RNA-Seq studies. In support of this we found that for the 12 candidates we investigated further these RNAs were found almost exclusively in the nuclear fraction. One point of note is that in this approach, purely because we exclude candidates which overlap annotated genes, we overlook antisense and gene-overlapping lncRNAs. By inspection, such RNAs are still immediately obvious, the *Kcnq1ot1* transcript being one example (Figure S12). Future experiments using strand-specific methodologies will help further annotate this part of the nuclear transcriptome [103], [104]. The nuclear-retained non-coding transcripts we identified are relatively stable and show lower association with RNAPII compared to other protein-coding genes expressed at similar levels (they are in the T sub-group). This suggests that they would be less easily identified using genome-wide techniques that identify nascent transcripts such as the GRO-Seq, NET-Seq and genome-wide nuclear run-on assays [9], [95], [105].

The accurate and thorough characterization of transcriptional output represents an important step in the understanding of the regulatory environment in which gene expression occurs for a particular cell type or induced state [106]. Sequencing the nuclear transcriptome reveals the relative levels of primary transcripts and in addition identifies novel nuclear retained lncRNAs not identified from total RNA-Seq studies. In this study we have presented a detailed description of the nuclear transcriptome in erythroid cells, though the methods described here could be applied to any given cell type or state including disease, experimentally perturbed states and cell fate changes.

## Methods

### Tissue collection

We collected spleens of anemic mice (C57BL/6) 5 days after treating them with phenylhydrazine [107]. The 5 day anemic spleen was found to be composed of >85% globin-expressing erythroid cells [40]. We disrupted fresh spleen tissue into a single-cell suspension in ice-cold phosphate-buffered saline and processed cells immediately as detailed below. All animal experimental procedures were carried out under a project license granted from the Home Office, UK.

### RNAPII ChIP-Seq

RNAPII ChIP was carried out as described in Mitchell and Fraser 2008 [59]. Genome-wide RNAPII association was determined by sequencing libraries constructed from the RNAPII-S5P chromatin immunopurified (using Ab5131, Abcam) and genomic input material.

### nucRNA-Seq

Genome-wide transcriptional output was characterized by sequencing a double-stranded cDNA library constructed from nuclear RNA (nucRNA). Following a hyper-osmotic swell in 10 mM Tris-HCl pH 7.5, 10 mM NaCl, 3 mM MgCl_2_, 0.1 M sucrose, 0.1% Triton X-100 and 0.5 mM DTT, a single cell suspension was homogenised with a B-type Dounce. Intact nuclei were then separated from cytoplasmic debris through a 5 mM MgCl_2_, 10 mM Tris pH 8.0, 0.5 mM DTT, 0.33 M sucrose cushion at 300 g and re-suspended in 10 mM Tris-HCl pH 7.5, 10 mM NaCl, 3 mM MgCl_2_. RNA was purified from nuclear and cytoplasmic fractions using Trizol LS (Invitrogen) according to the supplier's instructions. Purified RNA was treated with 10 U of DNaseI (Roche) for 20 min at 28°C. RNA quality was verified on a Bioanalyzer (Agilent). Reverse transcription was performed using Superscript II (Invitrogen) and 10 µg random hexamer primers (Roche) per 500 ng RNA. Second strand synthesis was performed using *E. coli* RNase H (Ambion) and *E. coli* DNA Polymerase I (NEB) as described in Sambrook and Russell 2001 [108].

### Sequencing and Data Analysis

Library preparation was performed according to Illumina PE genomic protocol, incorporating improvements suggested in Quail *et al* 2008, with all reactions scaled according to starting DNA quantity [109], [110]. Using the Illumina GA-IIx platform, we sequenced paired-end 36 bp reads from the generated libraries. Sequencing data was submitted to the Sequence Read Archive (SRA, http://www.ebi.ac.uk/ena/data/view/ERP000702). Sequences were aligned using Bowtie [111], by suppressing alignments to only 1 best reportable alignment with a maximum number of 2 mismatches within 28 nucleotides of seed length in the high quality end. A gap width of 2500 bp was allowed between paired end reads. When comparing to G1E and G1E-ER4+E2 RNA-Seq data all reads were mapped as single end reads using the indicated Bowtie settings. Sequences were visualised using SeqMonk [66] and the UCSC genome browser. We used the mouse Ensembl gene annotations throughout (genome version NCBIM37). Genes smaller than 300 bp were excluded from the list of genes investigated in the RNAPII stalling section. Peaks were identified using SISSRs (p<0.001) [65] and SeqMonk [66]. Perl, Java and R were used for further data processing. SPSS (version 18) was used for statistical analysis as detailed in the text.

### Transcript stability assay

Tissue was obtained and disrupted as described above, cultured for 1 or 4 hours in Dulbecco's Modified Eagle Medium (Gibco) supplemented with 10% fetal bovine serum and 10 µg/ml Actinomycin D (Sigma) with gentle mixing. Nuclear RNA and cDNA were prepared as detailed above. This cDNA was then used to assess the transcript stability in the absence of active transcription (primer sequences listed in Table S12).

### Real-time PCR

All RT-qPCR was carried out using SYBR Green on an ABI 7000 detection system (both Applied Biosystems). Primer sequences listed in Table S12.

### RNA FISH

RNA FISH was carried out as detailed in [112]. Probes were designed against intronic regions to detect primary transcripts. Expression was calculated as the percentage of alleles with a detectable signal in a cell population taken from randomly selected fields of view. Multiple probes were designed against candidate lncRNAs and were detected as for intronic probes.

## Supporting Information

Figure S1 **Reproducibility of nucRNA-Seq coverage.** RPKM values of nucRNA-Seq coverage in three biological replicate nucRNA-Seq libraries (F1.2, F2.2 and F3.2) are highly correlated (Spearman's rho >0.8, p<0.0001). Scales represent log2 RPKM values taken for Ensembl genes (genome version NCBIM37), *** indicates p<0.0001, correlation coefficients represent Spearman's rho.
(PDF)

Figure S2 **RT-qPCR Validation of nucRNA-Seq coverage for 48 amplicons.** Observed coverage in our sequence data for 48 randomly selected nucRNA-enriched regions was validated. For these regions, we assayed RNA levels by RT-qPCR in two independent nuclear RNA preparations. We observed a significant association between both the maximum nucRNA-Seq coverage depth (Spearman's rho (r_s_) = 0.761, 95% CI [0.608, 0.859], p<0.01) and average coverage depth (r_s_ = 0.781, 95% CI [0.638, 0.871], p<0.01).
(PDF)

Figure S3 **Normalising data coverage to input genomic DNA coverage.** A) A SeqMonk screenshot of a 0.5 Mb region around the *Hjurp* locus is depicted. Each track contains individual reads (small blue and red marks) and bars representing quantitated average coverage depth, non-normalised to input levels, for a 5 kb sliding window (1 kb step size). False positive enrichment of both nucRNA-Seq and RNAPII ChIP-Seq coverage can be observed around the *Hjurp* locus, in the area where input coverage is abnormally high. The need for normalisation is demonstrated by the fact that while clearly the *Hjurp* gene (centre, blue) is RNAPII bound and transcribed, it is not bound or transcribed at the levels indicated by non-normalised measures of coverage. (B and C) Shows a comparison of non-normalised RNAPII ChIP-Seq (B) and nucRNA-Seq (C) average coverage depth against the average input gDNA coverage depth for all annotated genes (NCBIM37), the middle panel shows a histogram of average coverage depth for annotated genes. The right histogram shows the same coverage normalised to the corresponding input value (fold enrichment over input).
(PDF)

Figure S4 **Nuclear RNA-Seq data compared to RNA-Seq data.** RPKM values for exon 1 were compared between erythroid nucRNA-Seq and two erythroid RNA-Seq (G1E and G1e_ER4_E2). The two RNA-Seq libraries are highly correlated (Spearman's rho 0.88) while the nucRNA-Seq library is less well correlated (Spearman's rho 0.25 and 0.30). Scales represent log2 RPKM values taken for Ensembl genes (genome version NCBIM37), *** indicates p<0.0001.
(PDF)

Figure S5 **Real-time PCR validation of RNAPII ChIP material.** Fold enrichment relative to input was determined for specific gene regions by real-time PCR. We detected reproducibly high levels of enrichment at erythroid-expressed genes (*Hba*, *Hbb*, *Slc4a1*, and *Hmbs*) while non-expressed genes (*Nefm and VH16*) were not enriched above background binding relative to the IgG control material or in relation to the input material. Error bars represent SEM calculated for 3 technical replicates.
(PDF)

Figure S6 **Nuclear RNA and RNAPII ChIP sequencing tag density at erythroid-expressed genes.** Sequence coverage at the A) *Hba* and B) *Slc4a1* genes.
(PDF)

Figure S7 **Validation of RNAPII ChIP-Seq coverage for 48 amplicons.** Observed coverage in our sequence data was validated for the same 48 randomly selected nucRNA-enriched regions used in Figure S3. For these regions, we assayed fold ChIP enrichment over input by qPCR in three independent RNAPII ChIP experiments. We observed a significant association between the fold enrichment assessed by qPCR and the RNAPII ChIP-Seq data, both for maximum coverage depth in the tested amplicon (r_s_ = 0.683, 95% CI [0.489, 0.812], p<0.01) and for average coverage depth (r_s_ = 0.668, 95% CI [0.477, 0.799], p<0.01).
(PDF)

Figure S8 **Stalling categories.** We compared promoter proximal and terminator proximal stalling, identifying 300 genes with promoter stalling, 300 genes with terminator (3′ end) stalling and 60 genes with both promoter and terminator (3′ end) stalling (thresholds set at the 95th percentile for each category).
(PDF)

Figure S9 **RNAPII ChIP-Seq coverage at genes in the promoter-proximal, 3′ end and double RNAPII peak categories.** A) *Calm1* displays a promoter-proximal RNAPII peak, B) *Sec14l2* displays a 3′ end RNAPII peak, C) *Pttg1ip* displays an RNAPII peak in both the promoter-proximal and 3′end region. Sequenced tags are depicted in black, fold enrichment over input in the promoter-proximal region (+/−300 bp), 3′ end (+/−300 bp) and gene body is shown by grey boxes with numbers indicating the fold enrichment value in each region. Image exported from SeqMonk.
(PDF)

Figure S10 **Putative regulatory regions upstream of erythroid expressed genes.** A) Two intergenic RNAPII peaks upstream of the *Pim1* gene overlap several TF binding sites. B) One RNAPII peak upstream of the Klf3 gene overlaps several TF binding sites.
(TIF)

Figure S11 **Stable ncRNA candidates expressed in erythroid cells.** Mouse chr19 is depicted from 5758468–5875817 (117 kbp) with annotated coding mRNA shown in red (forward) and blue (reverse) depending on the transcript direction. Candidate ncRNAs identified by Guttman et al 2009 are indicated by dark grey boxes. Candidate ncRNAs identified in our study are indicated by light grey boxes. NucRNA sequences are depicted below the ncRNA candidates. Image exported from SeqMonk.
(TIF)

Figure S12 **The** ***Kcnq1ot1*** **ncRNA is detected by nucRNA-Seq.** Mouse chr7 is depicted from 150293116–150612579 (319.46 kbp). *Kcnq1* transcripts are depicted with the nucRNA sequences mapped to this region depicted below. The region of increased nucRNA levels corresponds to the antisense *Kcnq1ot1* trasncript. Image exported from SeqMonk.
(TIF)

Table S1 **Number of reads per kilobase of gene length per million mapped reads (RPKM) in nucRNA-Seq replicates.**
(XLSX)

Table S2 **Transcription frequency determined by RNA FISH.**
(DOC)

Table S3 **Gene Ontology term enrichments for B, T and BT gene classes.**
(XLSX)

Table S4 **RNAPII binding patters, promoter peak, terminator peak and double peaks.**
(XLSX)

Table S5 **RNAPII+/nucRNA- peaks.**
(XLSX)

Table S6 **Transcription factor ChIP-Seq data used.**
(DOC)

Table S7 **Overlap between ChIP-Seq peaks.** Using 1 kb bins across the genome overlapping regions of RNAPII+/nucRNA- and all erythroid transcription factors (TFs) or p300 were investigated. Log odds ratios and P values were calculated for peaks in each of the indicated regions of the genome.
(DOC)

Table S8 **Validation of supervised motif analysis; indentified motifs for RNAPII+/nucRNA- candidates overlapped by TF binding sites identified through publicly available ChIP-Seq data.**
(DOC)

Table S9 **Supervised motif analysis for RNAPII+/nucRNA- candidates not overlapped by TF binding sites identified through publicly available ChIP-Seq data.**
(DOC)

Table S10 **Predicted ncRNA candidates.**
(XLSX)

Table S11 **Selected long ncRNA candidate regions.**
(DOC)

Table S12 **RNAPII ChIP-seq and nuRNA-seq validation primers.**
(DOC)
