# Supplementary figures and images for: Nuclear RNA Sequencing of the Mouse Erythroid Cell Transcriptome
Source: PLoS One. 2012 Nov 29;7(11):e49274. doi: 10.1371/journal.pone.0049274 (PMC3510205; doi:10.1371/journal.pone.0049274)

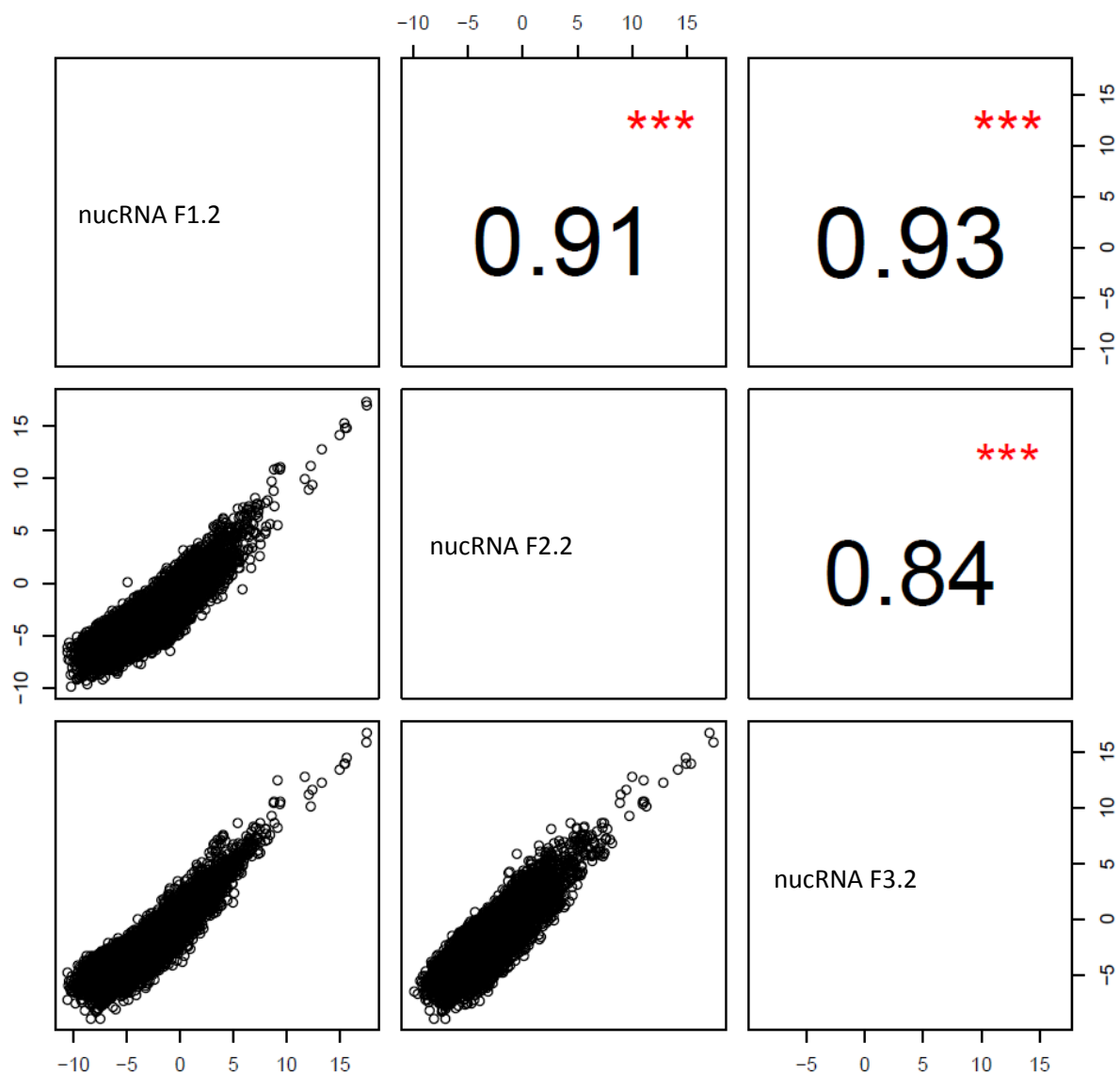

Supplement: Figure S1 — Reproducibility of nucRNA-Seq coverage. RPKM values of nucRNA-Seq coverage in three biological replicate nucRNA-Seq libraries (F1.2, F2.2 and F3.2) are highly correlated (Spearman's rho >0.8, p<0.0001). Scales represent log2 RPKM values taken for Ensembl genes (genome version NCBIM37), *** indicates p<0.0001, correlation coefficients represent Spearman's rho. (PDF) [file pone.0049274.s001.pdf]

nucRNA-seq amplicon coverage

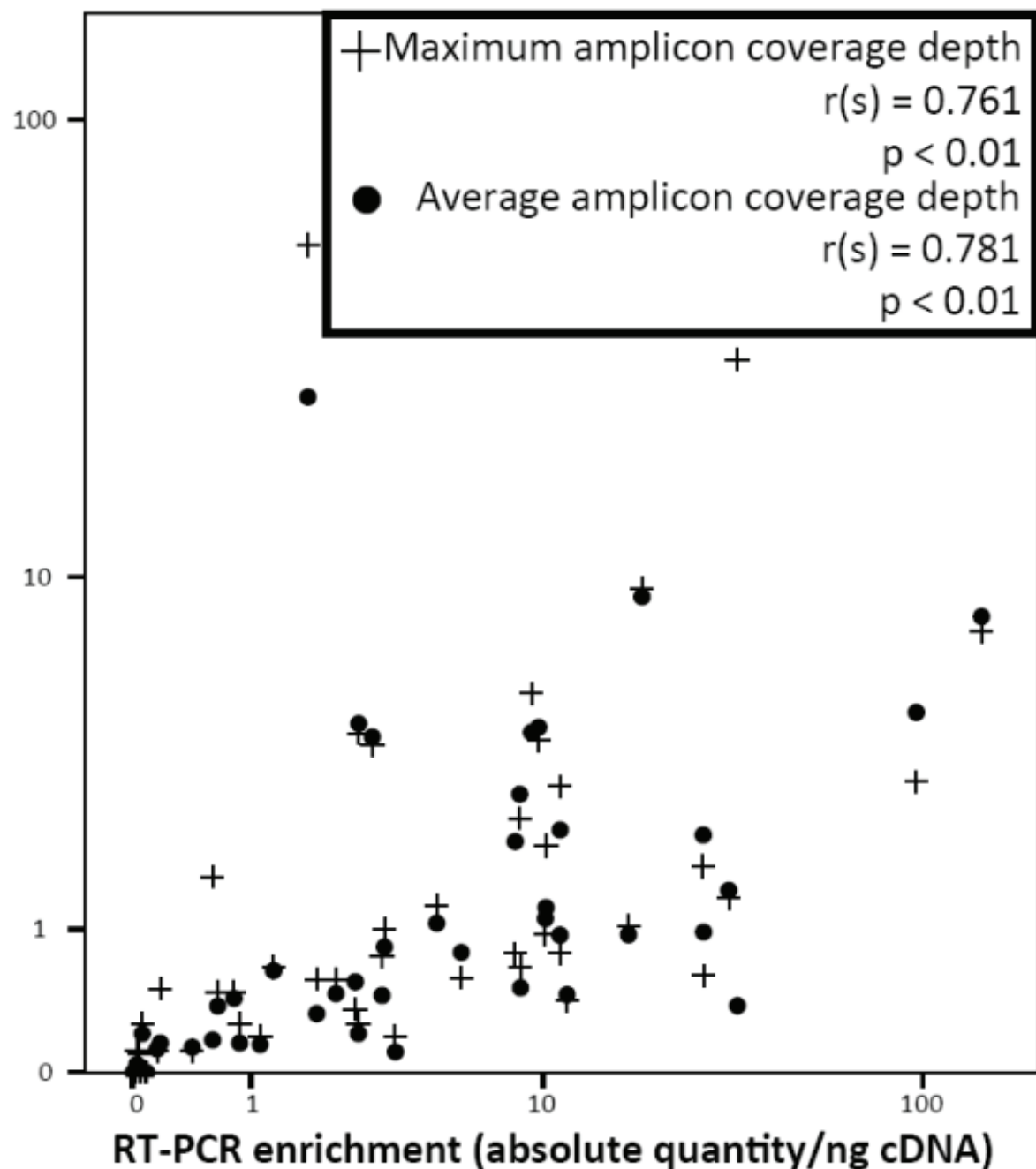

Supplement: Figure S2 — RT-qPCR Validation of nucRNA-Seq coverage for 48 amplicons. Observed coverage in our sequence data for 48 randomly selected nucRNA-enriched regions was validated. For these regions, we assayed RNA levels by RT-qPCR in two independent nuclear RNA preparations. We observed a significant association between both the maximum nucRNA-Seq coverage depth (Spearman's rho (rs) = 0.761, 95% CI [0.608, 0.859], p<0.01) and average coverage depth (rs = 0.781, 95% CI [0.638, 0.871], p<0.01). (PDF) [file pone.0049274.s002.pdf]

**A**

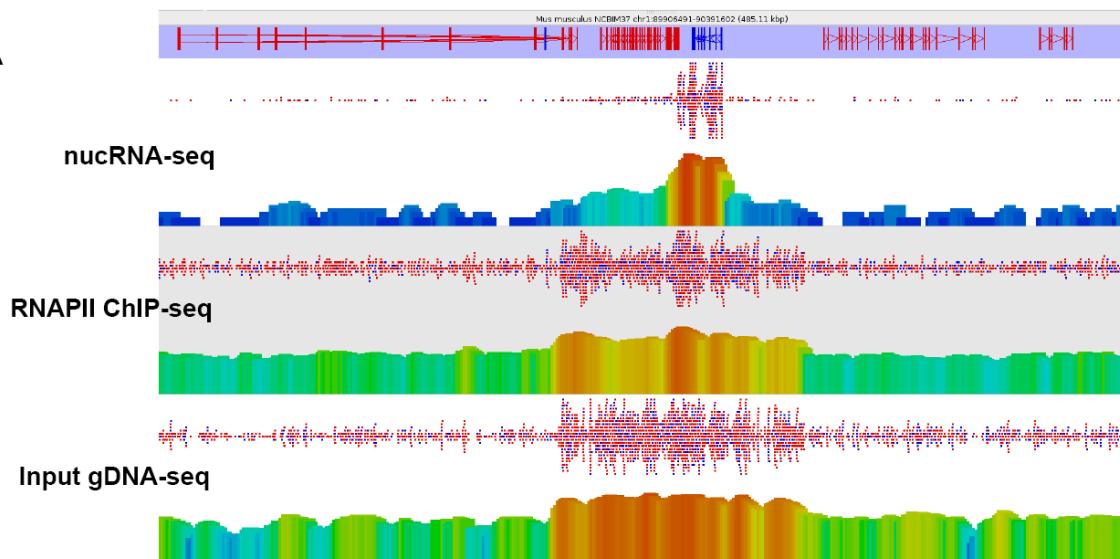

**B**

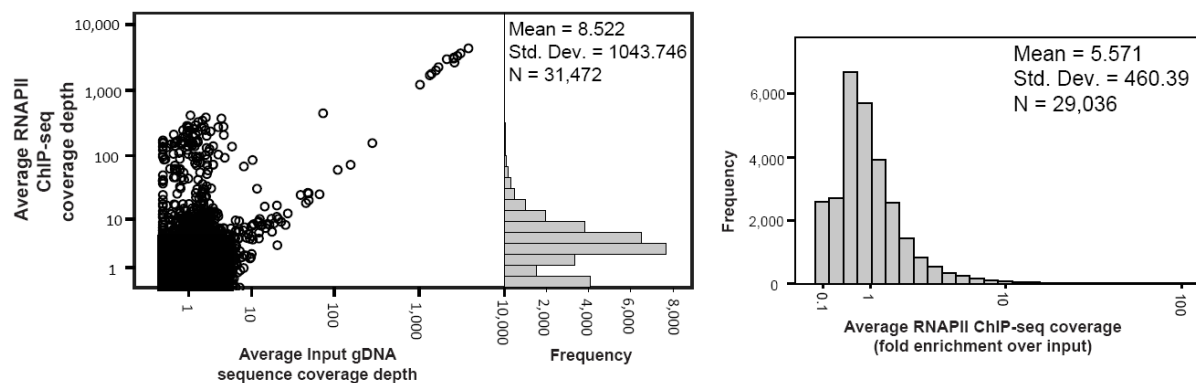

**C**

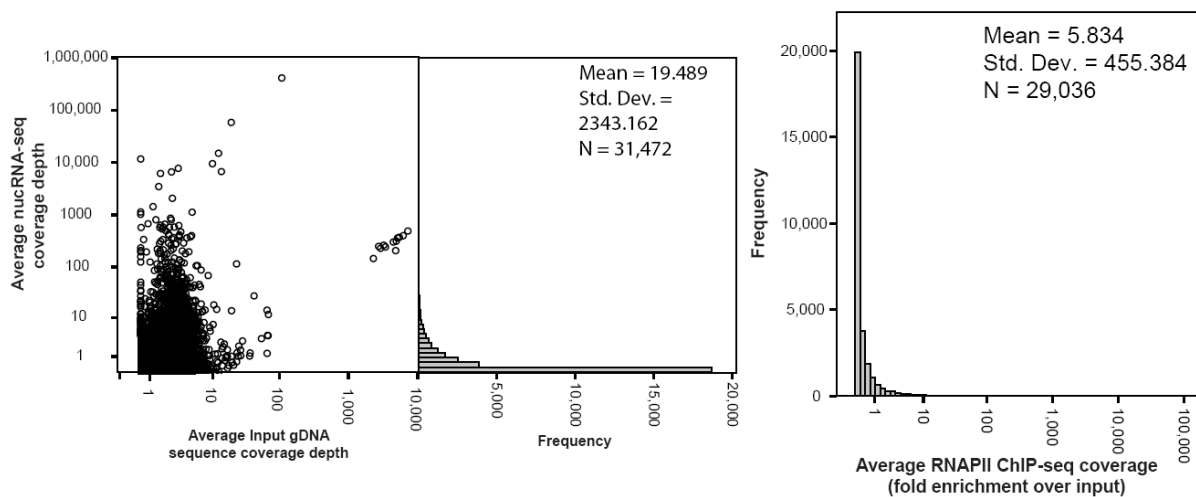

Supplement: Figure S3 — Normalising data coverage to input genomic DNA coverage. A) A SeqMonk screenshot of a 0.5 Mb region around the Hjurp locus is depicted. Each track contains individual reads (small blue and red marks) and bars representing quantitated average coverage depth, non-normalised to input levels, for a 5 kb sliding window (1 kb step size). False positive enrichment of both nucRNA-Seq and RNAPII ChIP-Seq coverage can be observed around the Hjurp locus, in the area where input coverage is abnormally high. The need for normalisation is demonstrated by the fact that while clearly the Hjurp gene (centre, blue) is RNAPII bound and transcribed, it is not bound or transcribed at the levels indicated by non-normalised measures of coverage. (B and C) Shows a comparison of non-normalised RNAPII ChIP-Seq (B) and nucRNA-Seq (C) average coverage depth against the average input gDNA coverage depth for all annotated genes (NCBIM37), the middle panel shows a histogram of average coverage depth for annotated genes. The right histogram shows the same coverage normalised to the corresponding input value (fold enrichment over input). (PDF) [file pone.0049274.s003.pdf]

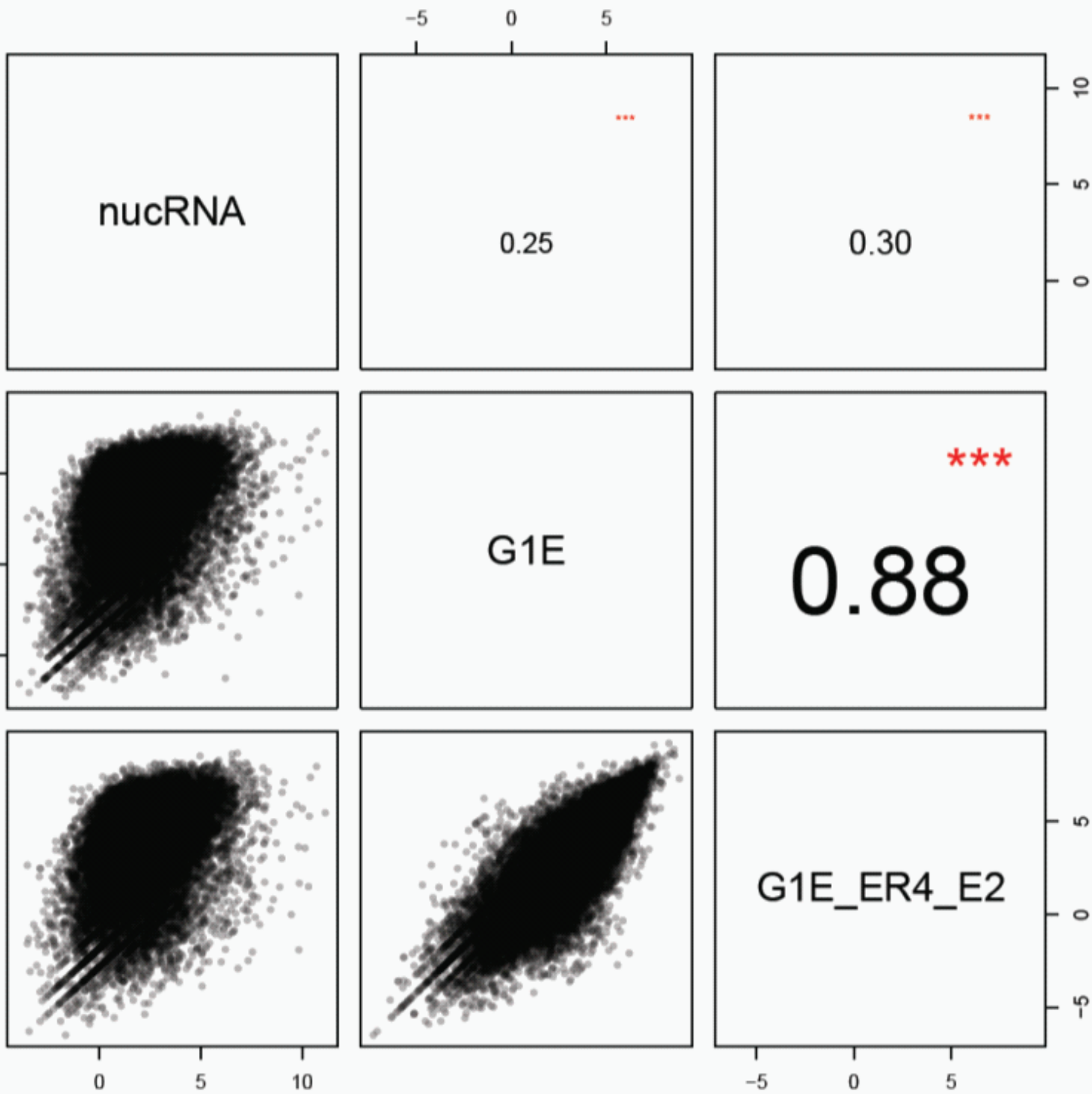

Supplement: Figure S4 — Nuclear RNA-Seq data compared to RNA-Seq data. RPKM values for exon 1 were compared between erythroid nucRNA-Seq and two erythroid RNA-Seq (G1E and G1e_ER4_E2). The two RNA-Seq libraries are highly correlated (Spearman's rho 0.88) while the nucRNA-Seq library is less well correlated (Spearman's rho 0.25 and 0.30). Scales represent log2 RPKM values taken for Ensembl genes (genome version NCBIM37), *** indicates p<0.0001. (PDF) [file pone.0049274.s004.pdf]

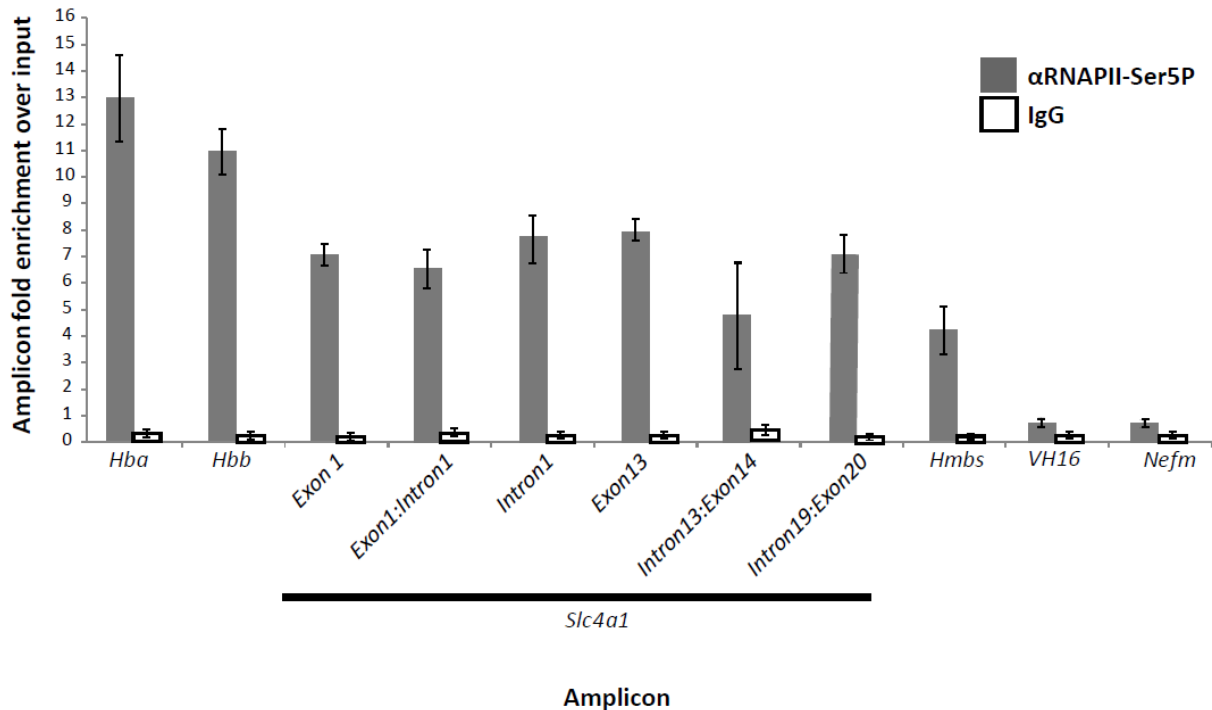

Supplement: Figure S5 — Real-time PCR validation of RNAPII ChIP material. Fold enrichment relative to input was determined for specific gene regions by real-time PCR. We detected reproducibly high levels of enrichment at erythroid-expressed genes (Hba, Hbb, Slc4a1, and Hmbs) while non-expressed genes (Nefm and VH16) were not enriched above background binding relative to the IgG control material or in relation to the input material. Error bars represent SEM calculated for 3 technical replicates. (PDF) [file pone.0049274.s005.pdf]

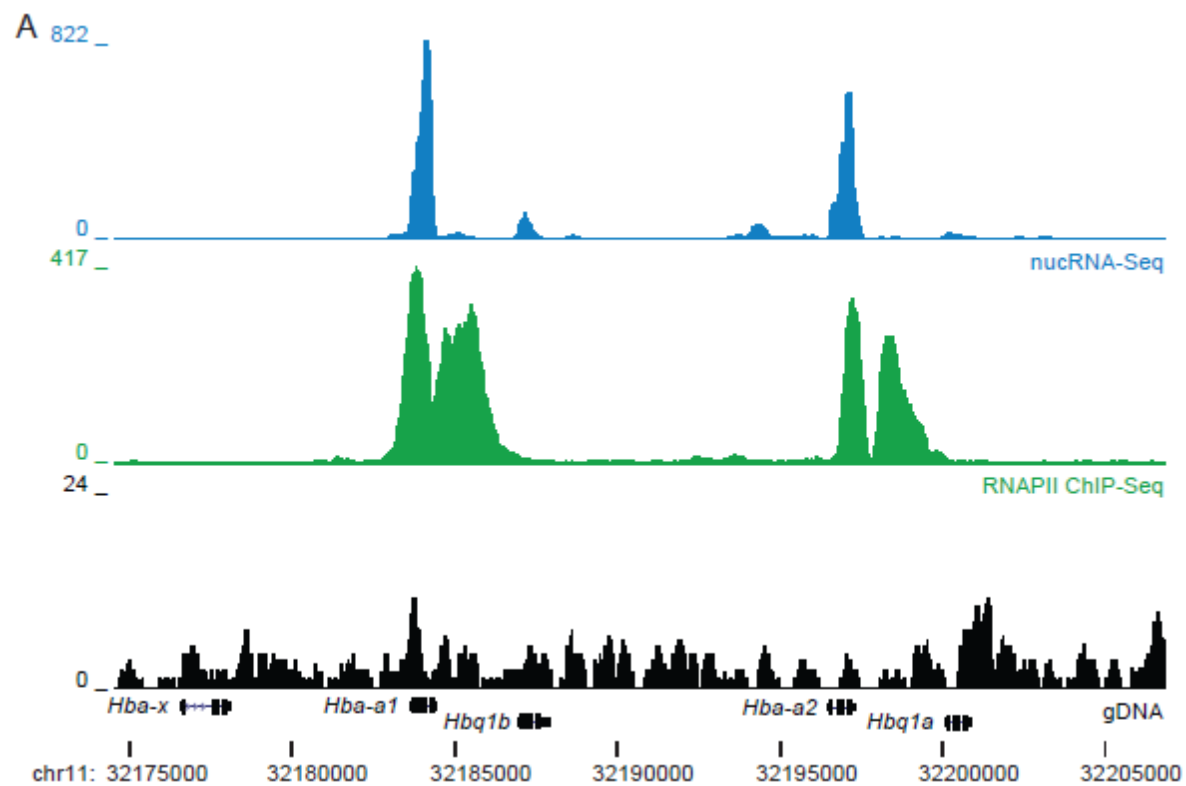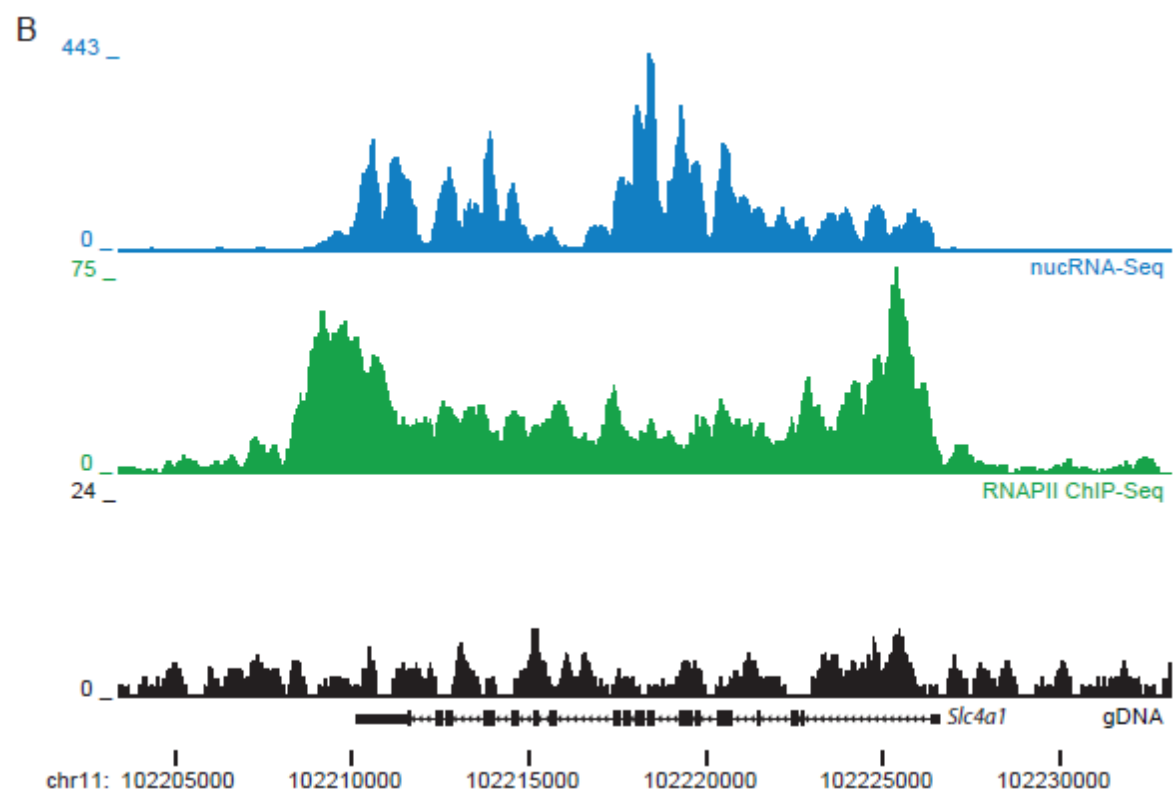

Supplement: Figure S6 — Nuclear RNA and RNAPII ChIP sequencing tag density at erythroid-expressed genes. Sequence coverage at the A) Hba and B) Slc4a1 genes. (PDF) [file pone.0049274.s006.pdf]

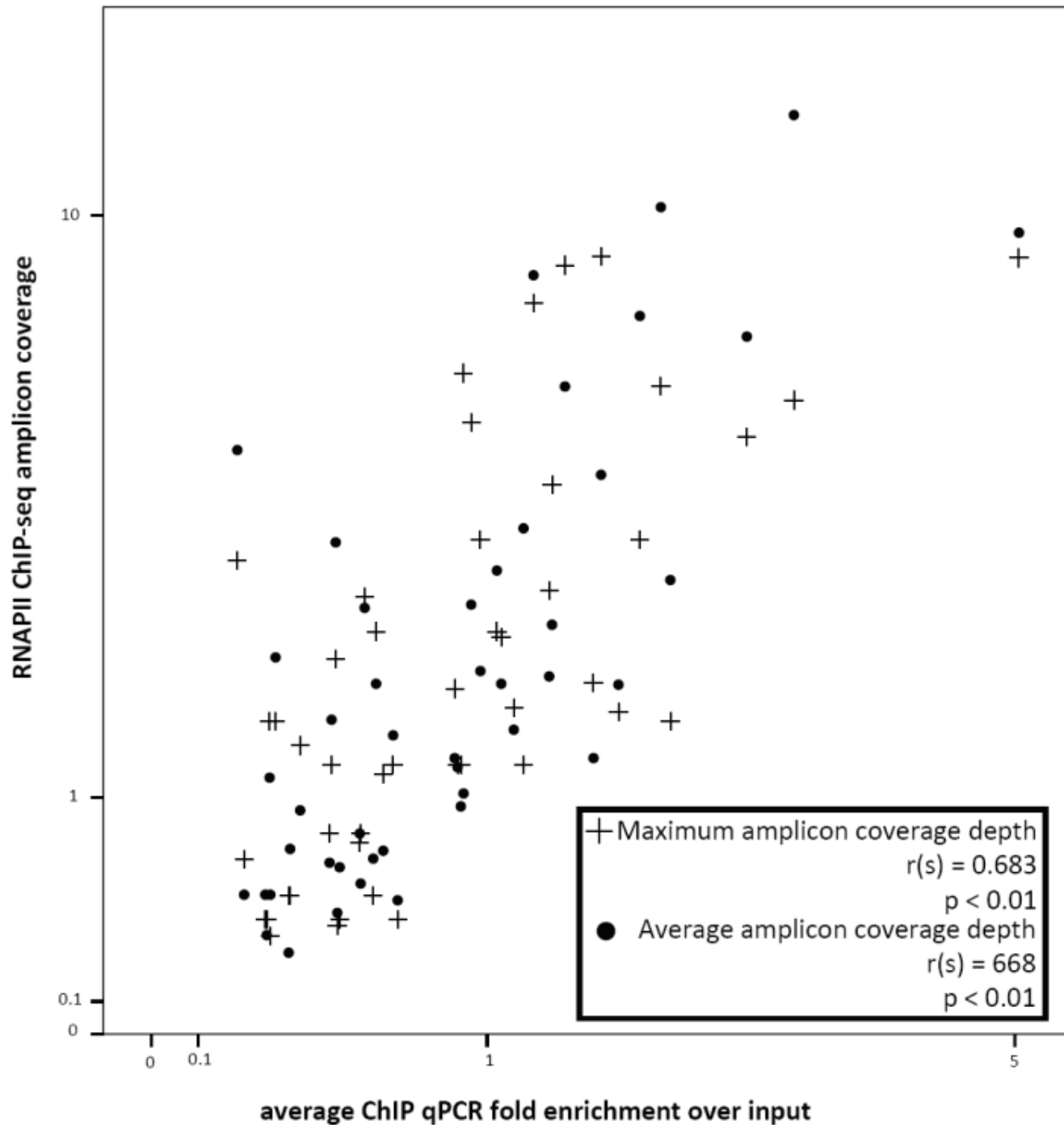

Supplement: Figure S7 — Validation of RNAPII ChIP-Seq coverage for 48 amplicons. Observed coverage in our sequence data was validated for the same 48 randomly selected nucRNA-enriched regions used in Figure S3. For these regions, we assayed fold ChIP enrichment over input by qPCR in three independent RNAPII ChIP experiments. We observed a significant association between the fold enrichment assessed by qPCR and the RNAPII ChIP-Seq data, both for maximum coverage depth in the tested amplicon (rs = 0.683, 95% CI [0.489, 0.812], p<0.01) and for average coverage depth (rs = 0.668, 95% CI [0.477, 0.799], p<0.01). (PDF) [file pone.0049274.s007.pdf]

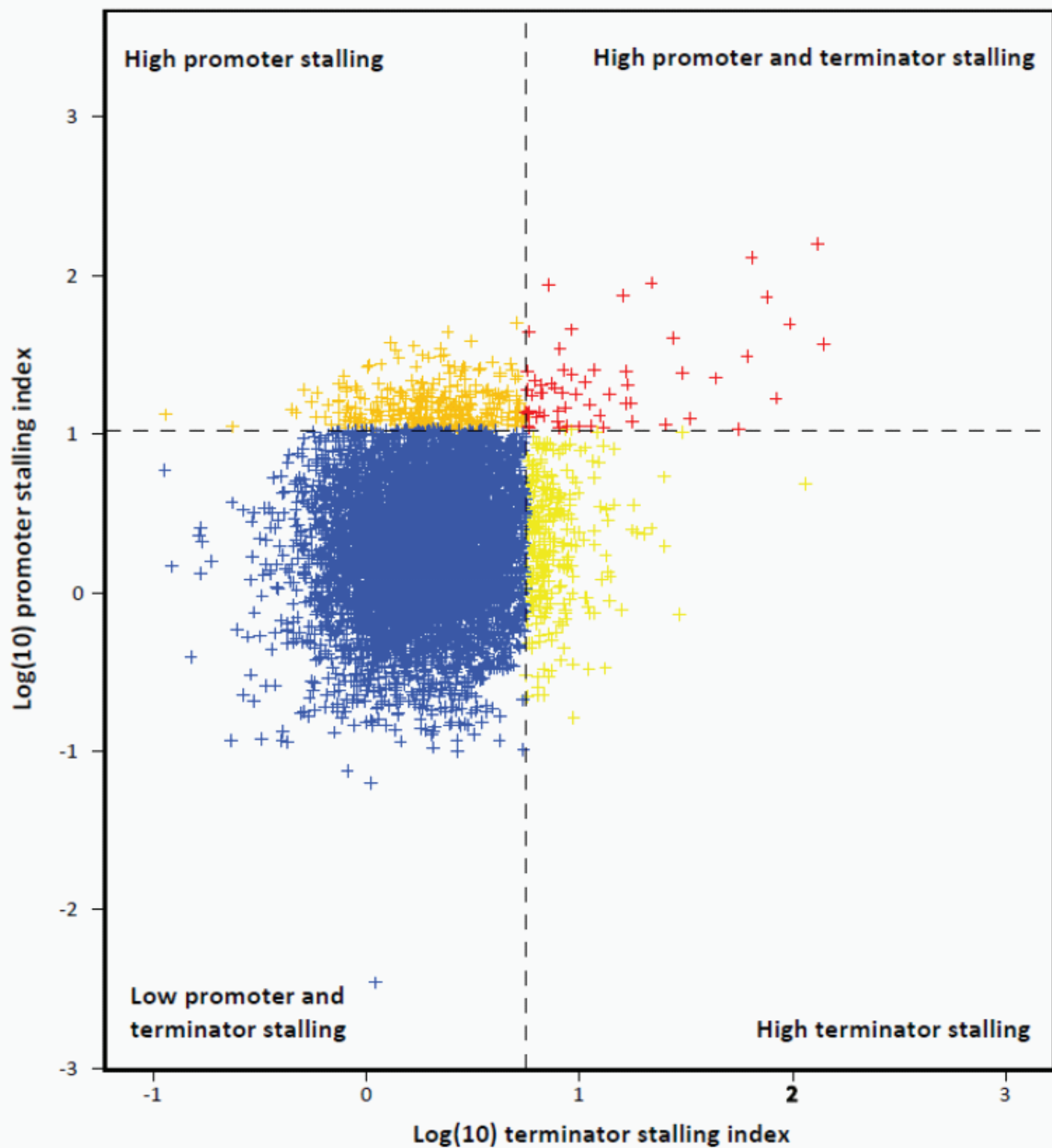

Supplement: Figure S8 — Stalling categories. We compared promoter proximal and terminator proximal stalling, identifying 300 genes with promoter stalling, 300 genes with terminator (3′ end) stalling and 60 genes with both promoter and terminator (3′ end) stalling (thresholds set at the 95th percentile for each category). (PDF) [file pone.0049274.s008.pdf]

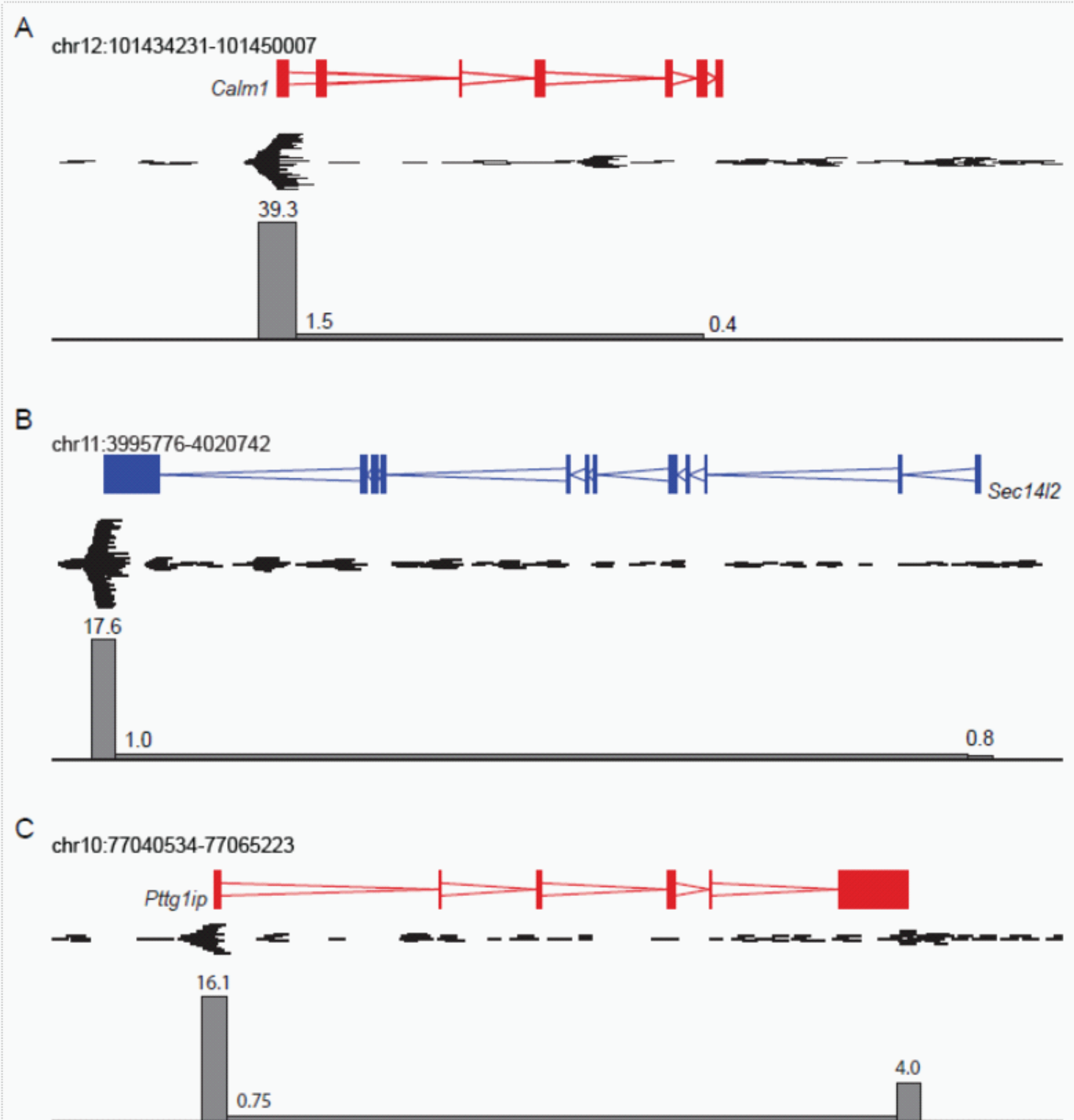

Supplement: Figure S9 — RNAPII ChIP-Seq coverage at genes in the promoter-proximal, 3′ end and double RNAPII peak categories. A) Calm1 displays a promoter-proximal RNAPII peak, B) Sec14l2 displays a 3′ end RNAPII peak, C) Pttg1ip displays an RNAPII peak in both the promoter-proximal and 3′end region. Sequenced tags are depicted in black, fold enrichment over input in the promoter-proximal region (+/−300 bp), 3′ end (+/−300 bp) and gene body is shown by grey boxes with numbers indicating the fold enrichment value in each region. Image exported from SeqMonk. (PDF) [file pone.0049274.s009.pdf]

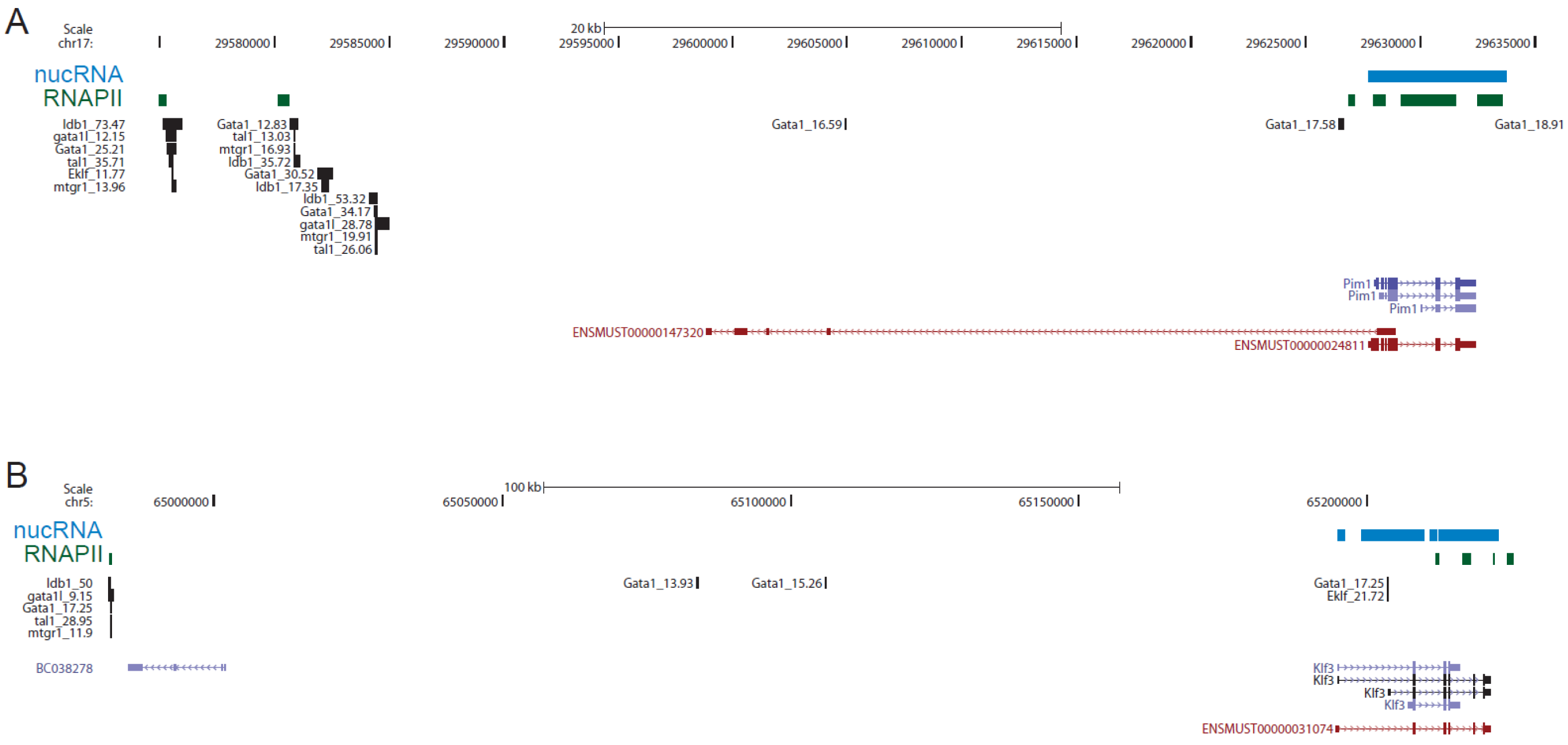

Supplement: Figure S10 — Putative regulatory regions upstream of erythroid expressed genes. A) Two intergenic RNAPII peaks upstream of the Pim1 gene overlap several TF binding sites. B) One RNAPII peak upstream of the Klf3 gene overlaps several TF binding sites. (TIF) [file pone.0049274.s010.tif]

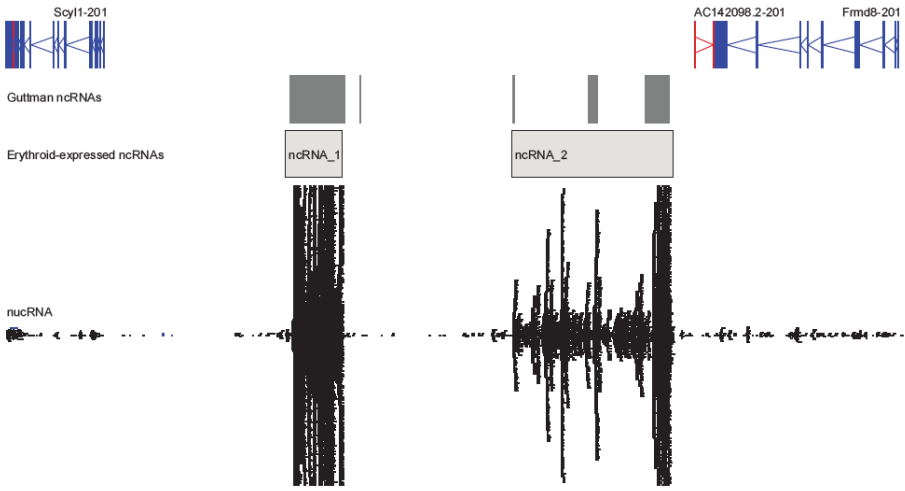

Supplement: Figure S11 — Stable ncRNA candidates expressed in erythroid cells. Mouse chr19 is depicted from 5758468–5875817 (117 kbp) with annotated coding mRNA shown in red (forward) and blue (reverse) depending on the transcript direction. Candidate ncRNAs identified by Guttman et al 2009 are indicated by dark grey boxes. Candidate ncRNAs identified in our study are indicated by light grey boxes. NucRNA sequences are depicted below the ncRNA candidates. Image exported from SeqMonk. (TIF) [file pone.0049274.s011.tif]

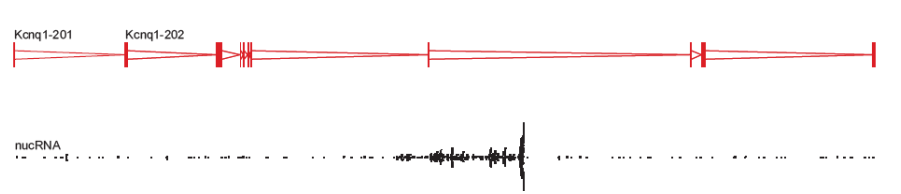

Supplement: Figure S12 — The Kcnq1ot1 ncRNA is detected by nucRNA-Seq. Mouse chr7 is depicted from 150293116–150612579 (319.46 kbp). Kcnq1 transcripts are depicted with the nucRNA sequences mapped to this region depicted below. The region of increased nucRNA levels corresponds to the antisense Kcnq1ot1 trasncript. Image exported from SeqMonk. (TIF) [file pone.0049274.s012.tif]
