# Supplementary material for: Nuclear RNA Sequencing of the Mouse Erythroid Cell Transcriptome
Source: PLoS One. 2012 Nov 29;7(11):e49274. doi: 10.1371/journal.pone.0049274 (PMC3510205; doi:10.1371/journal.pone.0049274)
Supplement: Table S2 — Transcription frequency determined by RNA FISH. (DOC) [file pone.0049274.s014.doc]

| **Gene name** | **ensEMBL id** | **maximum nucRNA-seq coverage** | **Transcription frequency (%)** |
| --- | --- | --- | --- |
| ***Abo*** | ENSMUSG00000015787 | 0.30 | 36.00 |
| ***Ank1*** | ENSMUSG00000031543 | 4.48 | 36.00 |
| ***Car1*** | ENSMUSG00000027556 | 0.57 | 35.00 |
| ***Car2*** | ENSMUSG00000027562 | 23.89 | 56.00 |
| ***Cpox*** | ENSMUSG00000022742 | 8.88 | 54.00 |
| ***Epb4.1*** | ENSMUSG00000028906 | 16.07 | 56.00 |
| ***Epb4.2*** | ENSMUSG00000023216 | 3.21 | 40.00 |
| ***Fech*** | ENSMUSG00000024588 | 11.23 | 41.00 |
| ***Gapdh*** | ENSMUSG00000057666 | 2.20 | 45.00 |
| ***Gypa*** | ENSMUSG00000051839 | 3.73 | 44.00 |
| ***Hmbs*** | ENSMUSG00000032126 | 19.75 | 45.00 |
| ***Nfe2*** | ENSMUSG00000058794 | 11.50 | 36.00 |
| ***Ppox*** | ENSMUSG00000062729 | 60.14 | 60.00 |
| ***Rhag*** | ENSMUSG00000023926 | 3.38 | 49.00 |
| ***Hba*** | ENSMUSG00000069919 | 172.82 | 86.15 |
| ***Hbb*** | ENSMUSG00000073940 | 424.67 | 84.62 |
| ***Band3*** | ENSMUSG00000006574 | 56.15 | 64.54 |
| ***Xpo7*** | ENSMUSG00000022100 | 12.64 | 63.44 |
| ***March3*** | ENSMUSG00000032656 | 26.14 | 62.14 |
