# Supplementary material for: Nuclear RNA Sequencing of the Mouse Erythroid Cell Transcriptome
Source: PLoS One. 2012 Nov 29;7(11):e49274. doi: 10.1371/journal.pone.0049274 (PMC3510205; doi:10.1371/journal.pone.0049274)
Supplement: Table S6 — Transcription factor ChIP-Seq data used. (DOC) [file pone.0049274.s018.doc]

| Antibody | Cell Type | Reference |
| --- | --- | --- |
| p300  p300 (SC-584) | MEL | ENCODE (M Snyder, Stanford University) |
| LDB1  & ETO2  & TAL1  & MTGR1  & GATA1 | MEL (differentiated) | Soler *et al* 2010 |
| GATA1 | G1E-ER4 cells | Cheng *et al* 2009 |
| EKLF/KLF1 | E14.5 fetal livers | Tallack *et al* 2010 |
