# Supplementary material for: Nuclear RNA Sequencing of the Mouse Erythroid Cell Transcriptome
Source: PLoS One. 2012 Nov 29;7(11):e49274. doi: 10.1371/journal.pone.0049274 (PMC3510205; doi:10.1371/journal.pone.0049274)
Supplement: Table S7 — Overlap between ChIP-Seq peaks. Using 1 kb bins across the genome overlapping regions of RNAPII+/nucRNA- and all erythroid transcription factors (TFs) or p300 were investigated. Log odds ratios and P values were calculated for peaks in each of the indicated regions of the genome. (DOC) [file pone.0049274.s019.doc]

| **Genome Regions** | **Overlap to TFs** | | **Overlap to p300** | |
| --- | --- | --- | --- | --- |
|  | **Log Odds** | **P value** | **Log Odds** | **P value** |
| All regions | 3.036 | <0.0001 | 1.6006 | <0.0001 |
| TSS (+/- 500bp) removed | 2.9676 | <0.0001 | 1.5401 | <0.0001 |
| TSS only | 1.7398 | <0.0001 | 1.0859 | <0.0001 |
| Genic | 2.8275 | <0.0001 | 1.5893 | <0.0001 |
| Intergenic | 3.1732 | not calc | 1.5312 | <0.0001 |
| upstream | 2.8372 | not calc | 1.7086 | <0.0001 |
| downstream | 2.1198 | not calc | 1.1689 | <0.0001 |
| >10kb | 3.3656 | not calc | 1.1222 | <0.0001 |
