# Supplementary material for: Nuclear RNA Sequencing of the Mouse Erythroid Cell Transcriptome
Source: PLoS One. 2012 Nov 29;7(11):e49274. doi: 10.1371/journal.pone.0049274 (PMC3510205; doi:10.1371/journal.pone.0049274)
Supplement: Table S8 — Validation of supervised motif analysis; indentified motifs for RNAPII+/nucRNA- candidates overlapped by TF binding sites identified through publicly available ChIP-Seq data. (DOC) [file pone.0049274.s020.doc]

| **Motif** | **Raw score** | **p-value relative to chr19** | **p-value relative to promoters (5kb upstream of TSS)** |
| --- | --- | --- | --- |
| Klf4 | 40.8 | 0 | 0 |
| TAL1::GATA1 | 31.9 | 0 | 0 |
| Gata1 | 27.1 | 0 | 0 |
| NFYA | 7.69 | 0.001 | 0.001 |
