# Supplementary material for: Nuclear RNA Sequencing of the Mouse Erythroid Cell Transcriptome
Source: PLoS One. 2012 Nov 29;7(11):e49274. doi: 10.1371/journal.pone.0049274 (PMC3510205; doi:10.1371/journal.pone.0049274)
Supplement: Table S9 — Supervised motif analysis for RNAPII+/nucRNA- candidates not overlapped by TF binding sites identified through publicly available ChIP-Seq data. (DOC) [file pone.0049274.s021.doc]

| **Motif** | **Raw score** | **P-value relative to chr19** | **p-value relative to promoters (5kb upstream of TSS)** |
| --- | --- | --- | --- |
| SPI1 | 133 | 0 | 0 |
| GABPA | 115 | 0 | 0 |
| NFYA | 78 | 0 | 0 |
| KLF4 | 73.2 | 0 | 0.002 |
| FEV | 62.9 | 0 | 0 |
| SPIB | 56.3 | 0 | 0 |
| ELF5 | 47.8 | 0.001 | 0 |
| ELK4 | 28.1 | 0 | 0 |
| ETS1 | 26.8 | 0 | 0 |
| ELK1 | 18.7 | 0 | 0 |
| ZFX | 10.1 | 0.003 | 0.003 |
| SRY | 8.73 | 0.001 | 0 |
