# Supplementary material for: Nuclear RNA Sequencing of the Mouse Erythroid Cell Transcriptome
Source: PLoS One. 2012 Nov 29;7(11):e49274. doi: 10.1371/journal.pone.0049274 (PMC3510205; doi:10.1371/journal.pone.0049274)
Supplement: Table S11 — Selected long ncRNA candidate regions. (DOC) [file pone.0049274.s023.doc]

| **Chr** | **Start (bp)** | **End (bp)** | **ID** | **Predicted Strand** |
| --- | --- | --- | --- | --- |
| 19 | 5,795,055 | 5,802,463 | 1_comp | -1 |
| 19 | 5,824,639 | 5,845,692 | 2_comp | -1 |
| 13 | 107,853,160 | 107,915,823 | 3_comp | ? |
| 14 | 62,245,729 | 62,303,919 | 4_comp | -1 |
| 12 | 32,781,760 | 32,808,472 | 9_comp | ? |
| 13 | 24,591,189 | 24,682,520 | 11_comp | ? |
| 14 | 61,252,924 | 61,317,072 | 12-1_comp | 1 |
| 14 | 61,252,924 | 61,317,072 | 12-2_comp | -1 |
| 9 | 13,245,402 | 13,276,333 | 13_comp | ? |
| 9 | 13,327,599 | 13,401,806 | 14_comp | ? |
| 12 | 81,217,507 | 81,233,860 | 16_comp | ? |
| 6 | 99,293,256 | 99,471,452 | 20_comp | -1 |
| 7 | 30,245,834 | 30,304,843 | 21_comp | ? |
