# Supplementary material for: Nuclear RNA Sequencing of the Mouse Erythroid Cell Transcriptome
Source: PLoS One. 2012 Nov 29;7(11):e49274. doi: 10.1371/journal.pone.0049274 (PMC3510205; doi:10.1371/journal.pone.0049274)
Supplement: Table S12 — RNAPII ChIP-seq and nuRNA-seq validation primers. (DOC) [file pone.0049274.s024.doc]

| *Name* | *Sequence 5’ to 3’* | *Notes* |
| --- | --- | --- |
| **HbaE1_FOR** | TTCTGACAGACTCAGGAAGAAACCA | Hba-a1 exon 1 |
| **HbaE1_REV** | AGCACCATGGCCACCAATCT |  |
| **HbaI2_FOR** | gacccctaggaagggcttgg | Hba-a1 intron 2-3 |
| **HbaI2_REV** | ggacaccctgatgcctctgc |  |
| **GapdhE1I1_FOR** | CTTCTTGTGCAGTGCCAGgtga | Gapdh exon 1-intron 1-2 |
| **GapdhE1I1_REV** | cgcaccagcatccctagacc |  |
| **slc4a1E1-F** | AGTTGGGAGCTCAGCCAGT | Slc4a1 Exon1 |
| **slc4a1E1-R** | GGTCCTTCGGGAAGTCCT |  |
| **Slc4a1E1I1_FOR** | TGGGAGCTCAGCCAGTCACA | Slc4a1 exon 1-intron 1-2 |
| **Slc4a1E1I1_REV** | cgggacagatgccaaaggac |  |
| **Slc4a1I1_FOR** | GATCTGAGGCCCAGCCAGAA | Slc4a1 intron 1-2 |
| **Slc4a1I1_REV** | CCCCACCTCCTTCACCTTCC |  |
| **slc4a1E13-F** | GCAGCTTCCTCGTCCAATAC | Slc4a1 Exon 13 |
| **slc4a1E13-R** | CAGGACTTGATCAGCTTGGAG |  |
| **slc4a1I13E14-F** | GGCATAGCGTGTCTGTGAGA | Slc4a1 Intron 13 - Exon14 |
| **slc4a1I13E14-R** | TTCATGGTTGTGCAAAGAGG |  |
| **Slc4a1I19E20_FOR** | CAGCAGGGCTTGTCCAGGTT | Slc4a1 intron 19-20-exon 20 |
| **Slc4a1I19E20_REV** | CACCGTCCAGCTAGAGGGGTAG |  |
| **Uros I1_FOR** | GGGCTCATTCTCCTAACCACTGGCTAC | Uros Intron 1 |
| **Uros I1_REV** | GCAACGGACTGATAACTTCTGGCTCAC |  |
| **Nef3E1_FOR** | CAGCACCGTGTCCTCCTCCT | Nef3 exon 1 |
| **Nef3E1_REV** | GGCTGAAGTCGAGGCTGCTC |  |
| **VH16 genic F** | GGAGGGTCCACTAAACTCTCTTG | V16 gene segment genic |
| **VH16 genic R** | GCATAGCCTTTTCCACTCTCATC |  |
| **Hmbs-F** | ACCCCTGGGCCTTGTCATTT | Hmbs (intron 1) |
| **Hmbs-R** | TGCCTGGGCAGGAGATAAGC |  |
| **Air_F** | ACTTTGACAGAACAATCGGCTCAG | Air |
| **Air_R** | GAACATTTGCAAAGGACAGTCGAG |  |
| **Myc ex1** | gctgtttgaaggctggatttcct | MycE1I1 |
| **Myc int1** | cgctacattcaagacgcagaaaga |  |
| **ncRNA_1_F** | CACTTGTGGGGAGACCTTGT | ncRNA 1 |
| **ncRNA_1_R** | GTTACCAGCCCAAACCTCAA |  |
| **ncRNA_2_F** | CCAAGCAACTGCATCTCAAA | ncRNA 2 |
| **ncRNA_2_R** | CGTGGGTTGATGGGAATAAC |  |
| **ncRNA_3_F** | CCATGAGCCTCGATTTCATT | ncRNA 3 |
| **ncRNA_3_R** | ATCAAACACTGGTCGCATGA |  |
| **ncRNA_4_F** | GGAGGAAAGCGACACAAGAG | ncRNA 4 |
| **ncRNA_4_R** | TTGAATGAGCCAGTGACAGC |  |
| **ncRNA_9_F** | GCTCCGGGATAGTCAATGAA | ncRNA 9 |
| **ncRNA_9_R** | TTTGTGGTCAGAAGCACGAG |  |
| **ncRNA_11_F** | ACCTTCTGCTGCTCCCTACA | ncRNA 11 |
| **ncRNA_11_R** | CCCTTGTGTCCCTCTTCGTA |  |
| **ncRNA_12_F** | CGAGAAAGGCTGAGATGGAC | ncRNA 12 |
| **ncRNA_12_R** | AAGCCTCCTCCACAGCACTA |  |
| **ncRNA_13_F** | CTTGTGCACACTGTTGCTGA | ncRNA 13 |
| **ncRNA_13_R** | CATGCTGCCAGAAACAGAAA |  |
| **ncRNA_14_F** | CTCTTTTCCAGCCTGTTTGC | ncRNA 14 |
| **ncRNA_14_R** | AAAGCACCAGGAAGCACACT |  |
| **ncRNA_16_F** | GAAAGAGAAACCGCTCATGC | ncRNA 16 |
| **ncRNA_16_R** | GGTGCTTTCCCAAATTCTCA |  |
| **ncRNA_20_F** | TGGCAGCAGGTACTTCTGTG | ncRNA 20 |
| **ncRNA_20_R** | AGGCTTTGTGCTTGAGGAAA |  |
| **ncRNA_21_F** | CAATGACAGTGGCATCCATC | ncRNA 21 |
| **ncRNA_21_R** | TTTGTGGGATCCTCTTCCTG |  |
| **1** | GCCATCTGAGGGAATTTCAA | 1/2(chr3:84688996) |
| **2** | ATCCCTCTTGTCAGCCCTCT |  |
| **3** | TCCTGCAGAAACAGAGAGCA | 3/4(chr4:48440526) |
| **4** | CCCCACCAAAACCTAAATGA |  |
| **5** | GCGTGTCTGGCTCAGGTTAT | 5/6(chr7:117918580) |
| **6** | CGGAGGGGTTTGTATCACTG |  |
| **7** | CAGGCTCAAACAACAGCAAA | 7/8(chr9:60138016) |
| **8** | TCTCCGACTGATTGGTCCTC |  |
| **9** | AAAACAGGTGCAAAGCCACT | 9/10(chr11:62996139) |
| **10** | GGGACAGGGACCGTTAAGTT |  |
| **11** | CCAGTCAGAGATGGACAGCA | 11/12(chr11:86130647) |
| **12** | TGTTTCCTTATGCCATTTGC |  |
| **13** | GCAATTTCATGCCTTTGCTT | 13/14(chr14:74279655) |
| **14** | ACCCCCAAATCCTTCTTCAC |  |
| **15** | TGGATGTTCTCAGGGAAGTTG | 15/16(chr17:43122315) |
| **16** | GCGGTGTGGTAGTTTTAGCC |  |
| **17** | TGGTACACTGCAGCCTTTTG | 17/18(chr18:55216194) |
| **18** | TAGATGCCAGCATCATCAGC |  |
| **19** | CCCAGGGATCAAACTTGGTT | 19/20(chr19:4761463) |
| **20** | ATGACCAAGTTCTGGGGTTG |  |
| **21** | CTGGCAGCAAAGGCTAACAT | 21/22(chr2:30270838) |
| **22** | AGCAGTGGCGCAGTCTTTAC |  |
| **23** | AGCACCCAGAAGCCAGACTA | 23/24(chr3:136554994) |
| **24** | CTCTGTCCCTGTTCCCGATA |  |
| **25** | AGCCCGCTAGCTTGATGTAG | 25/26(chr4:138907673) |
| **26** | TATTTATTGCTGGGGCCTTG |  |
| **27** | TCACAGAGCGCTGTAGGTGT | 27/28(chr6:88275093) |
| **28** | AGGCTGCCCATAGCTTTACA |  |
| **29** | TTACCAGAGTTGGCCGTACC | 29/30(chr8:74945603) |
| **30** | CGGCCCACTAAACTTACGAG |  |
| **31** | TGTGTACAGGACCGACTTGC | 31/32(chr9:77602956) |
| **32** | GTTTGCGGACAGATGTGAGA |  |
| **33** | GGGAACAGAAGGAATGCAGA | 33/34(chr11:60515139) |
| **34** | ACCATGTGGTCCTTCTCTGG |  |
| **35** | TCCAGGTTGGAAAAGCTAGG | 35/36(chr11:69668475) |
| **36** | CCTACAGCGACTGGAGAAGG |  |
| **37** | CCAGGTTACCAAGCAAGACC | 37/38(chr17:88022225) |
| **38** | GGCAGAAAACTTGCATTCCT |  |
| **39** | CTGATGGAGCATGAAGACGA | 39/40(chr19:8946498) |
| **40** | CTCCCAGTCAGGACCTTCAG |  |
| **41** | TGCACATCCACAGGGATAAA | 41/42(chr2:75526589) |
| **42** | CAGTCTGAGGCTTGCTTGTG |  |
| **43** | TTAGGTGGTGCCCTGGTATC | 43/44(chr3:51034140) |
| **44** | GATAACCACTGAGGCCATGC |  |
| **45** | CCCTCAGGGTACAACGAAAG | 45/46(chr3:137530049) |
| **46** | ACATGCTTGGCATGAATCAA |  |
| **47** | GCCTTCTAGACGGTCAGTGC | 47/48(chr3:153365733) |
| **48** | GGGCGGTTTGAACTTTGTTA |  |
| **49** | CTTCAGTCACTCGCTCCACA | 49/50(chr4:117828487) |
| **50** | CATAGCCTCCTCCACTCAGC |  |
| **51** | CTTCTCTGTCGCATTGCTTG | 51/52(chr6:8601608) |
| **52** | ATAGACCCGAGGGCAGAAGT |  |
| **53** | TTGAGGCTCATCTGATCGTG | 53/54(chr9:44278636) |
| **54** | AGGTGGCACACAAATGTTGA |  |
| **55** | AGATTTCCCAAAAGCCCTGT | 55/56(chr13:58491594) |
| **56** | CCTGGCTTTATTGGGTGCTA |  |
| **57** | TGGATCCTGAGCACTCACAG | 57/58(chr15:30766893) |
| **58** | GCATGTGCCCCTTCATTACT |  |
| **59** | CCAATCAGAGCCTTCAGGAG | 59/60(chr18:90749114) |
| **60** | TCACACAGGACCACTCAAGC |  |
| **61** | CTGAAGATGCCACCAATCCT | 61/62(chr2:158603897) |
| **62** | TAACCGACAGGCTTGTACCC |  |
| **63** | ACTTTCCCCATTCTGGGATT | 63/64(chr7:24487568) |
| **64** | GTTCGCTGCACAGCAGTAAG |  |
| **65** | CCCTACCTCTGCAGTGAAGC | 65/66(chr7:139965812) |
| **66** | CGTTTTGCCCAAAGTGAAAT |  |
| **67** | ACCGAATTGCTGAGTGTTCC | 67/68(chr11:68604137) |
| **68** | GGCCAACACCAACCTTCTAA |  |
| **69** | TTTCTCCCTTTCCGGAGATT | 69/70(chr11:69571925) |
| **70** | TTCTTCCTCCCTCCCAAAAC |  |
| **71** | GGGCGTGTACACCTTCTGAT | 71/72(chr12:77726015) |
| **72** | AGGAGAAGGGGAATCTGGAA |  |
| **73** | GATGCCCTAGGACAGAACCA | 73/74(chr15:73578258) |
| **74** | CACCCAGCTCTCCTGAAGTC |  |
| **75** | GCAGCGCCTCGAGTAAATAA | 75/76(chr16:56076176) |
| **76** | TGGAGGCTGGAAAAGAAAAA |  |
| **77** | CCCTTCAAATGGTAGCGTGT | 77/78(chr17:15631543) |
| **78** | GTCAGGAATCCGTTTCTCCA |  |
| **79** | TCCAAGGAGAAAGGAAGCAA | 79/80(chr18:34212371) |
| **80** | TCTGTTACGGTGACCCTTCC |  |
| **81** | CCAGGCTTTGTAGTCGTGGT | 81/82(chr1:75138502) |
| **82** | TGCTGATTGAGTGCGTCTTC |  |
| **83** | GGGAGTGTGCCATCAAGTTT | 83/84(chr1:134250923) |
| **84** | ATCGGCCAGTAAGAATGGTG |  |
| **85** | CACGGGAGAAAGCAGAGAAC | 85/86(chr2:163456633) |
| **86** | CCTGACGACTGCACAGAAAA |  |
| **87** | AACTCGGCTTTAGCAGTGGA | 87/88(chr4:133370581) |
| **88** | AGTCAAGGAAGCGCAGTCAT |  |
| **89** | GCAAAGTCTCTTCCCCTTCC | 89/90(chr9:35017357) |
| **90** | TGCTTTGGACTTTGGAGCTT |  |
| **91** | TGTGCTGGTGCTGAGCTAAC | 91/92(chr11:84633149) |
| **92** | GAACAGATGGCCAAACCACT |  |
| **93** | GTAAGCCCGGAGACATTTCA | 93/94(chr13:23654460) |
| **94** | TTCTGGGAACATGCTTTGTG |  |
| **95** | AAAGGTACGTGATGGCCTTG | 95/96(chr13:29011486) |
| **96** | GCTGAAGACGATTGGGAAAA |  |
| **97** | GGTGGAGGAACGATGTGTTT | 97/98(chr13:55425693) |
| **98** | CAGCCTCTTACCTGGACAGC |  |
| **99** | AAGCTGAGTTGCCATGTGTG | 99/100(chr17:26059666) |
| **100** | ATGGACTTCTTGGCCTTTCC |  |
